# Poly-γ-glutamic acid/Alum adjuvanted pH1N1 vaccine-immunized aged mice exhibit a significant increase in vaccine efficacy with a decrease in age-associated CD8^+^ T cell proportion in splenocytes

**DOI:** 10.1186/s12979-022-00282-z

**Published:** 2022-05-23

**Authors:** Jihyun Yang, Jaemoo Kim, Chaewon Kwak, Haryoung Poo

**Affiliations:** 1 Infectious Disease Research Center, Korea Research Institute of Bioscience and Biotechnology (KRIBB), Daejeon, 34141 Republic of Korea; 2 Department of Biosystems and Bioengineering, KRIBB School of Biotechnology, University of Science and Technology (UST), Daejeon, 34113 Republic of Korea

**Keywords:** Aging, Vaccine adjuvant, Influenza virus, γ-PGA, CD8^+^ T lymphocyte, Dendritic cells

## Abstract

**Background:**

Highly contagious respiratory diseases caused by viral infections are a constantly emerging threat, particularly the elderly with the higher risk of developing serious complications. Vaccines are the best strategy for protection against influenza-related diseases. However, the elderly has lower vaccine efficacy than young population and the age-driven decline of the influenza vaccine efficacy remains unresolved.

**Objectives:**

This study investigates the effect of an adjuvant, poly-γ-glutamic acid and alum (PGA/Alum) on vaccine efficacy in aged mice (18-months) and its mechanism is investigated using ovalbumin as a model antigen and a commercial pandemic H1N1 (pH1N1) flu vaccine. Antigen trafficking, dendritic cell (DC) activation, and the DC-mediated T cell activation were analyzed via in vivo imaging and flow cytometry. Antigen-specific humoral and cellular immune responses were evaluated in sera and splenocytes from the vaccinated mice. Also, we analyzed gene expression profiles of splenocytes from the vaccinated mice via single-cell transcriptome sequencing and evaluated the protective efficacy against pH1N1 virus challenge.

**Results:**

Aged mice had lower antigen trafficking and DC activation than younger mice (6-weeks), which was ameliorated by PGA/Alum with increased antigen uptake and DC activation leading to improved antigen-specific IFN-γ^+^CD8^+^ T lymphocyte frequencies higher in the vaccinated aged mice, to a similar extent as PGA/Alum adjuvanted vaccine-immunized young mice. The results of single-cell transcriptome sequencing display that PGA/Alum also reduced the proportion of age-associated CD8^+^ T cell subsets and gene levels of inhibitory regulators in CD8^+^ T cells, which may play a role in the recovery of CD8^+^ T cell activation. Finally, PGA/Alum adjuvanted pH1N1 vaccine-immunized aged mice were completely protected (100% survival) compared to aged mice immunized with vaccine only (0% survival) after pH1N1 virus challenge, akin to the efficacy of the vaccinated young mice (100% survival).

**Conclusions:**

PGA/Alum adjuvanted pH1N1 vaccine-immunized aged mice showed a significant increase in vaccine efficacy compared to aged mice administered with vaccine only. The enhanced vaccine efficacy by PGA/Alum is associated with significant increases of activation of DCs and effector CD8^+^ T cells and a decrease in age-associated CD8^+^ T cell proportion of splenocytes. Collectively, PGA/Alum adjuvanted flu vaccine may be a promising vaccine candidate for the elderly.

**Supplementary information:**

The online version contains supplementary material available at 10.1186/s12979-022-00282-z.

## Background

Viral respiratory infections are highly contagious and rapidly spread through airborne transmission especially the pandemic influenza virus emergence poses a threat to the global public health [1]. In particular, young children, pregnant women, immunocompromised individuals, and the elderly are at risk associated with complicating infectious diseases [2]. Notably, the booming global population of individuals over the age of 65 is more susceptible to viral infection than the younger population [3]. Flu is a well-known annually recurring infectious respiratory disease caused mainly by the influenza A virus, accounting for almost 90% of seasonal flu-related deaths and 70% of seasonal flu-related hospitalizations in the elderly [4]. The healthcare cost associated with treating these infections is escalating and hence finding an effective strategy to combat viral infection in the elderly remains essential. Vaccination is considered one of the best strategies against viral infection, but the protective efficacy of the flu vaccines is still lower in the elderly than in the younger adults [5].

Aging affects physiological functions, particularly immune systems and leads to immunosenescence, a process of decreasing the ability to mount an immune response, increasing the vulnerability and severity to infectious diseases, and diminished responses to vaccination in the elderly [6–8]. Dendritic cells (DCs) are the most potent antigen-presenting cells bridging innate and adaptive immunities. Impaired ability of the DCs is therefore a prime culprit for reducing the vaccine efficacy [9]. Antigens are taken up by the DCs for processing and presenting the antigen peptides on the major histocompatibility complex (MHC) classes to be recognized by T cells in the draining lymph nodes (dLNs). During aging, antigen uptake by the DCs is reportedly lower than that of their young counterparts in humans [10], rats [11], and mice [12]. In addition, the DCs from aged mice show lower ability to prime CD4^+^ T lymphocytes [13] and cross-prime cytotoxic CD8^+^ T lymphocytes (CTLs) than the DCs from the young mice [12, 14]. The age-driven reduction of vaccine efficacy is also accountable to the deteriorated adaptive immunity, including low T cell numbers and their insufficient responses [15]. In particular, the CD8^+^ CTLs responsible for directly clearance of virus-infected cells are potent for devising vaccine strategies; however, their responses diminish gradually with aging. Several studies have reported the changes of CD8^+^ T cell subsets in aging, a decrease in the naive subset (CD44^−^) and an increase in the age-associated subset (CD44^+^ PD-1^+^) harboring the features of dysfunctional T cells. The age-associated CD8^+^ T cell subset has insufficient proliferation ability despite exposure to the antigens, antigen-exposed DCs, or polyclonal stimulation via anti-CD3 and anti-CD28 antibodies [16, 17]. Moreover, the dysfunctional CD8^+^ T cell subset is accompanied by increased expression of negative regulators for T cell responses including PD-1, LAG3, and Tox [18–20].

To date, flu vaccines for the elderly have been approved at a high dose of vaccine antigen or using an adjuvant to enhance the immune responses [21, 22]. Nevertheless, developing tailored vaccines remains an important issue due to the distinct immunological characteristics observed during aging. The significance of vaccines for the elderly depends on the vaccine formulations considering the implication of the aging immune system. Efforts have been made to develop adjuvants capable of increasing immunogenicity of vaccine antigen in aging [23], but the mechanism of action for recovering the age-driven decline of vaccine efficacy remains elusive. An adjuvant has been previously developed by combining the poly-γ-glutamic acid (γ-PGA) with the alum called PGA/Alum [24]. The γ-PGA is a safe and edible biomaterial secreted naturally by *Bacillus subtilis* that induces the innate immune responses through Toll-like receptor (TLR) 4 signaling, enhancing the cellular immunity [25], and alum is a well-known licensed adjuvant triggering humoral immunity [26]. In young mice, PGA/Alum robustly increased antigen trafficking and DC activation, resulting in the adaptive immune responses, particularly CTL activity [24]. The protective immunity against the pandemic H1N1 (pH1N1) flu vaccine was improved in the young mice, suggesting that PGA/Alum may recover the efficacy of the flu vaccine in aged mice.

Here, the impact of PGA/Alum on the efficacy of the flu vaccine in aged mice (18-months) is investigated by comparing with the young mice (6-weeks), and mechanisms of action were elucidated using ovalbumin (OVA) as a model antigen and a commercial pH1N1 flu vaccine. Antigen trafficking, migration, and activation of the DCs and the DC-mediated T cell activation were analyzed via in vivo imaging and flow cytometry. The adjuvanticity of PGA/Alum was analyzed in immunization with OVA or flu vaccine in aged mice via flow cytometry, enzyme-linked immunospot (ELISPOT), and enzyme-linked immunosorbent assay (ELISA). Also, we monitored expression profiles of splenocytes from the vaccinated aged mice using single-cell RNA sequencing (scRNA-seq) and finally evaluated the protective efficacy against homologous viral challenge. We demonstrate that the use of PGA/Alum as an adjuvant recovers the age-driven low efficacy of the vaccine via enhancement of antigen-loaded DC migration and DC-mediated CD8^+^ T cells activation, followed by more robust antigen-specific cellular responses with decreases of age-associated CD8^+^ T cell subsets and inhibitory molecules within CD8^+^ T cells, ultimately leading to full protection against influenza virus in aged mice. These findings have a range of implications for design of optimal vaccine platforms for the elderly.

## Results

### Age-driven declined abilities of antigen trafficking, DC migration and activation, and DC-mediated T cell activation are ameliorated by PGA/Alum

**Fig. 1:**
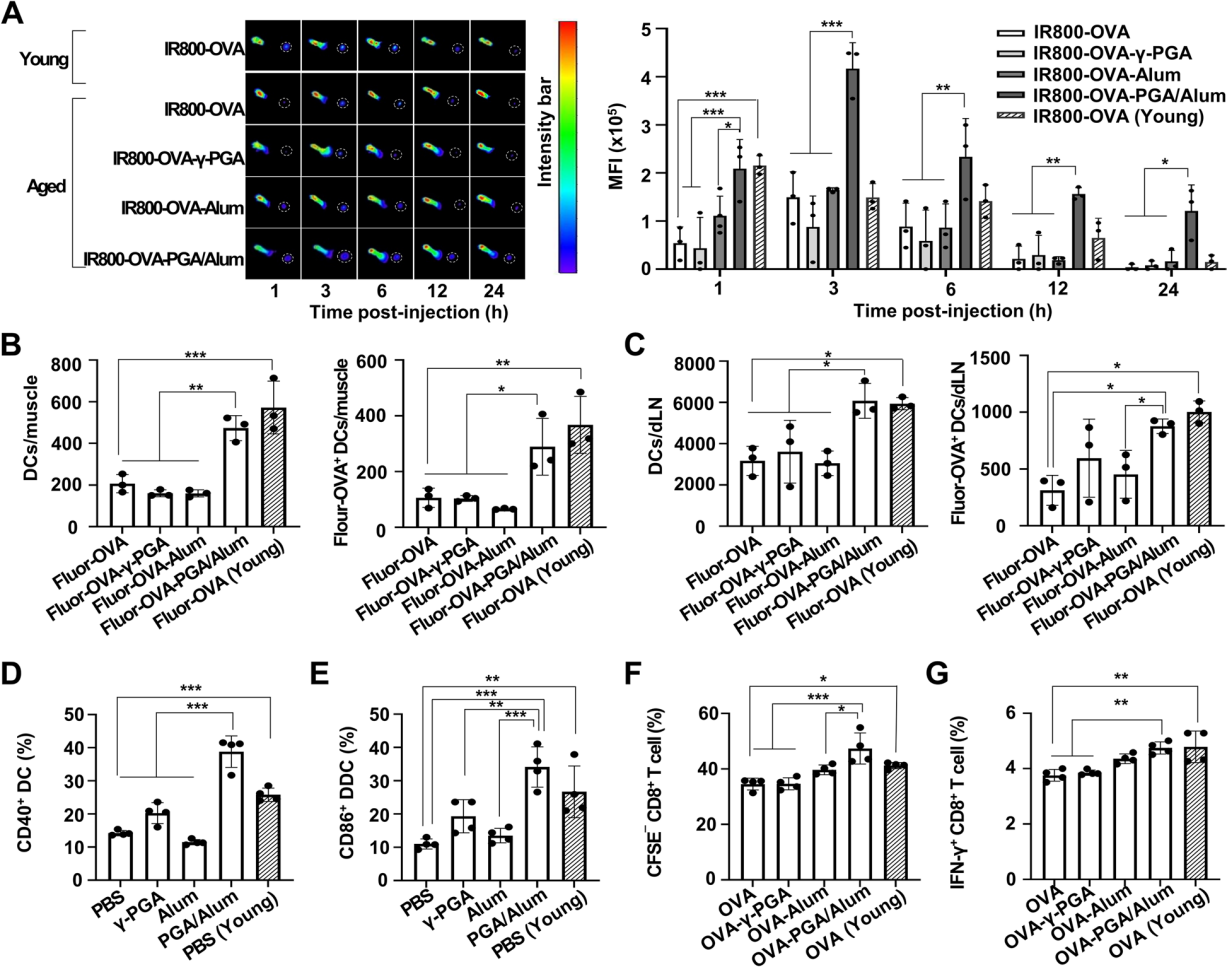
PGA/Alum recovers low DC migration, activation, and DC-mediated CD8^+^ T cell activation in aged mice. **A** Aged mice (*n* = 3 per group) were s.c. injected into the right footpad with IR800-OVA alone or mixed with γ-PGA, alum, or PGA/Alum. IR800-OVA-young mice (*n* = 3) were used for comparison. At 1, 3, 6, 12, and 24 h, in vivo NIR fluorescence signals were acquired using in vivo imaging system. Fluorescent intensities of LN of interest (dotted circle) were quantitatively measured using ImageJ. **B** and **C** Aged mice (*n* = 3 per group) were intramuscularly (i.m.) administered Fluor-OVA alone or mixed with γ-PGA, alum, or PGA/Alum. Fluor-OVA-young mice (*n* = 3) were used for comparison. At 3 h post-immunization, the number of DCs (gated as CD11c^+^CD3^−^) and Fluor-OVA^+^ DCs were analyzed in injected muscle region (**B)** and dLNs (**C)** via flow cytometry. **D–G** Splenic DCs from aged mice were stimulated with γ-PGA, alum, or PGA/Alum. (**D** and **E**) After 30 h, the DCs were stained with a fluorescent dye-conjugated anti-CD11c, CD40, and CD86 Abs. Expression of the costimulatory molecules was analyzed in CD11c^+^ DCs via flow cytometry. (**F** and **G)** After 6 h, the DCs were washed and co-cultured with CFSE-labeled OT-I CD8^+^ T cells for 5 days (**F**) or OT-I CD8^+^ T cells in the presence of monensin for 6 h  (**G**). Statistical significance was analyzed using two-way ANOVA/Bonferroni (**A**) and one-way ANOVA/Bonferroni (**B**–**G**); *P < 0.05, **P < 0.01, and ***P < 0.001

For inducing the adaptive immunity, DCs should facilitate antigen trafficking to dLNs, antigen processing and presentation, and upregulation of the co-stimulatory molecules and cytokines [9]. Similar to previous studies [12, 27], we compared the in vivo antigen trafficking in the aged (18-months) and young (6-weeks) mice (*n* = 3 per group) using OVA via the near-infrared (NIR) fluorescence imaging system. Mice were subcutaneously (s.c.) administered IRDye800-labeled OVA (IR800-OVA), and in vivo fluorescent signals were observed at 1, 3, 6, 12, and 24 h post-injection. As shown in Fig. 1A, IR800-OVA-young mice exhibited robust fluorescent signals in dLNs at 1, 3, and 6 h post-injection, and the fluorescent signals gradually decreased until 24 h post-injection. In dLNs of IR800-OVA-aged mice, however, the fluorescent signals were weak at 1 h and peaked at 3 h post-injection only. The mean fluorescence intensities (MFIs) used for quantitative measurements were also highest in the dLNs of IR800-OVA-young mice 1 h post-injection (215,625 MFI), whereas the MFIs in the dLNs of the IR800-OVA-aged mice were fourfold lower (53,916 MFI) than those of the young mice 1 h post-injection (*P* < 0.001), suggesting delayed antigen trafficking in the aged mice compared to the young mice. To determine whether PGA/Alum restores antigen trafficking in aging, aged mice were s.c. administered IR800-OVA or combined with γ-PGA (IR800-OVA-γ-PGA), alum (IR800-OVA-Alum), or PGA/Alum (IR800-OVA-PGA/Alum). IR800-OVA-PGA/Alum-aged mice had at least twofold higher MFIs in the dLN than other aged groups at all the time points (*P* < 0.05). Notably, MFIs of the IR800-OVA-PGA/Alum-aged mice were similar to those of the IR800-OVA-young mice, 1 h post-injection. The robust MFIs were significantly twofold higher than those of the IR800-OVA-young mice until 3 to 24 h post-injection.

To determine whether PGA/Alum-increased antigen trafficking in aged mice was due to the migration of antigen-loaded DCs, aged mice (*n* = 3 per group) were i.m. injected with Alexa Fluor 647-OVA (Fluor-OVA) alone or in combination with γ-PGA (Fluor-OVA-γ-PGA), alum (Fluor-OVA-Alum), and PGA/Alum (Fluor-OVA-PGA/Alum). The total DC and Fluor-OVA^+^ DC numbers were determined in injected muscle and dLNs 3 h post-injection via flow cytometry, and the Fluor-OVA-young mice were used for comparison. As expected, significantly lower numbers of DCs and Fluor-OVA^+^ DCs were observed in injected muscle of the Fluor-OVA-aged mice (206 and 106 cells) than the Fluor-OVA-young mice (624 and 401 cells) (*P* < 0.01) (Fig. 1B and Fig. S1). In the aged mice, the Fluor-OVA-PGA/Alum group exhibited twofold higher numbers of DCs and Fluor-OVA^+^ DCs (473 and 289 cells) than the other groups (206 and 106 cells in the Fluor-OVA; 160 and 104 cells in the Fluor-OVA-γ-PGA; 160 and 66 cells in Fluor-OVA-Alum) (*P* < 0.05). In addition, in the dLNs, significantly lower numbers of migrated DCs and OVA^+^ DCs were observed in the Fluor-OVA-aged mice (3,170 and 313 cells) than the Fluor-OVA-young mice (5,936 and 1,003 cells) (*P* < 0.05) (Fig. 1C and Fig. S2). However, the Fluor-OVA-PGA/Alum-aged mice showed increased numbers of DCs and OVA^+^ DCs (6,076 and 877 cells) compared to the other aged mice (3,170 and 313 cells in Fluor-OVA; 3,612 and 595 cells in Fluor-OVA-γ-PGA; 3,050 and 454 cells in the Fluor-OVA-Alum). Such effects were also observed in the Fluor-OVA-PGA/Alum-aged mice 12 h post-injection compared to the other aged mice (*P* < 0.05) (Fig. S3). These results indicate that PGA/Alum can restore the reduced DC migration ability observed in the aged mice.

Next, DC functions were examined using splenic CD11c^+^ DCs purified from the aged and young mice (*n* = 4 per group). Flow cytometry showed that the co-stimulatory molecules (CD40 and CD86) levels were significantly lower in the DCs from the aged mice (CD40; 14.7 ± 1.1% and CD86; 14.0 ± 2.4%) compared to those of young mice (CD40; 25.0 ± 1.4% and CD86; 35.5 ± 5.2%) (*P* < 0.001), but PGA/Alum (CD40; 48.0 ± 3.2% and CD86; 38.5 ± 4.5%) triggered highly their levels on DCs from the aged mice compared to PBS (CD40; 14.7 ± 1.1% and CD86; 14.0 ± 2.4%), γ-PGA (CD40; 21.0 ± 2.6% and CD86; 23.8 ± 5.6%), and alum (CD40; 15.0 ± 1.1% and CD86; 21.8 ± 3.5%) (*P* < 0.01) (Fig. 1D, E, and Fig. S4). In DCs from the aged mice, production of the inflammatory cytokines (IL-6, IFN-γ, TNF-α, IL-1α, MCP-1, and IL-1β) were also increased by PGA/Alum compared to PBS, γ-PGA, and alum (*P* < 0.05), although the quantity of γ-PGA in PGA/Alum is identical to γ-PGA alone (Fig. S5). To clarify whether the ability of PGA/Alum complex is affected by combination conditions, we compared the cytokine levels by DCs upon exposure to PGA/Alum complex or a simple mixture of γ-PGA and Alum and found that the PGA/Alum complex significantly increased the levels of TNF-α and IL-1β by DCs compared to the simple mixture (unpublished observation), suggesting that the conjugation process of PGA/Alum is important for enhancing innate immune responses. Moreover, the antigen uptake and processing of DCs were tested using FITC-OVA or DQ-OVA, which are well-established models for antigen uptake and processing, respectively. The percentages of FITC-OVA^+^ DCs and DQ-OVA^+^ DCs were lower in the DCs from the aged mice than in those from the young mice, and PGA/Alum increased the percentages compared to the γ-PGA or non-treatment when using DCs from the aged mice (*P* < 0.05) (Fig. S6). However, the small fold changes, even if statistically significant, were observed in the DCs of all the aged groups. Since the cross-presentation ability of DCs is a unique function to directly activate the CTLs capable of clearing the viral infection [9], the DC-mediated CD8^+^ T cell activation was assessed using the OVA-specific MHC class I (H-2K^b^)-restricted OT-I CD8^+^ T cells. CD11c^+^ DCs were incubated with OVA alone or combined with γ-PGA (OVA-γ-PGA), alum (OVA-Alum), and PGA/Alum (OVA-PGA/Alum) for 6 h, followed by co-culture with the OT-I CD8^+^ T cells. The T cell activation was analyzed using the CFSE-diluted profiles and IFN-γ production via flow cytometry. As shown in Fig. 1F and Fig. S7A, the percentages of CFSE^−^ CD8^+^ T cells were lower in co-culture with OVA-exposed DCs from the aged mice (27.1 ± 2.1%) than in the young mice (35.1 ± 1.5%), but were significantly higher in the co-culture with the OVA-PGA/Alum-exposed DCs (60.1 ± 4.5%) than the OVA (27.1 ± 2.1%), OVA-γ-PGA (33.2 ± 1.4%), or OVA-Alum-exposed DCs (50.3 ± 3.8%) (*P* < 0.001). The percentages of the IFN-γ^+^CD8^+^ T cells were also lower in the co-culture with OVA-exposed DCs from the aged mice (3.7 ± 0.1%) than in those from the young mice (4.7 ± 0.4%) (*P* < 0.001), but the age-driven low percentages were significantly higher in the co-culture with OVA-PGA/Alum-exposed DCs (4.7 ± 0.1%) than in the DCs exposed to OVA (3.7 ± 0.1%) or OVA-γ-PGA (3.8 ± 0.1%) (*P* < 0.01) (Fig. 1G and Fig. S7B). These results suggest that PGA/Alum can enhance the age-driven decline of antigen trafficking, DC migration and activation, and DC-mediated T cell activation.

### PGA/Alum robustly increases the antigen-specific CTL activity more effectively rather than antibody production in the aged mice

**Fig. 2:**
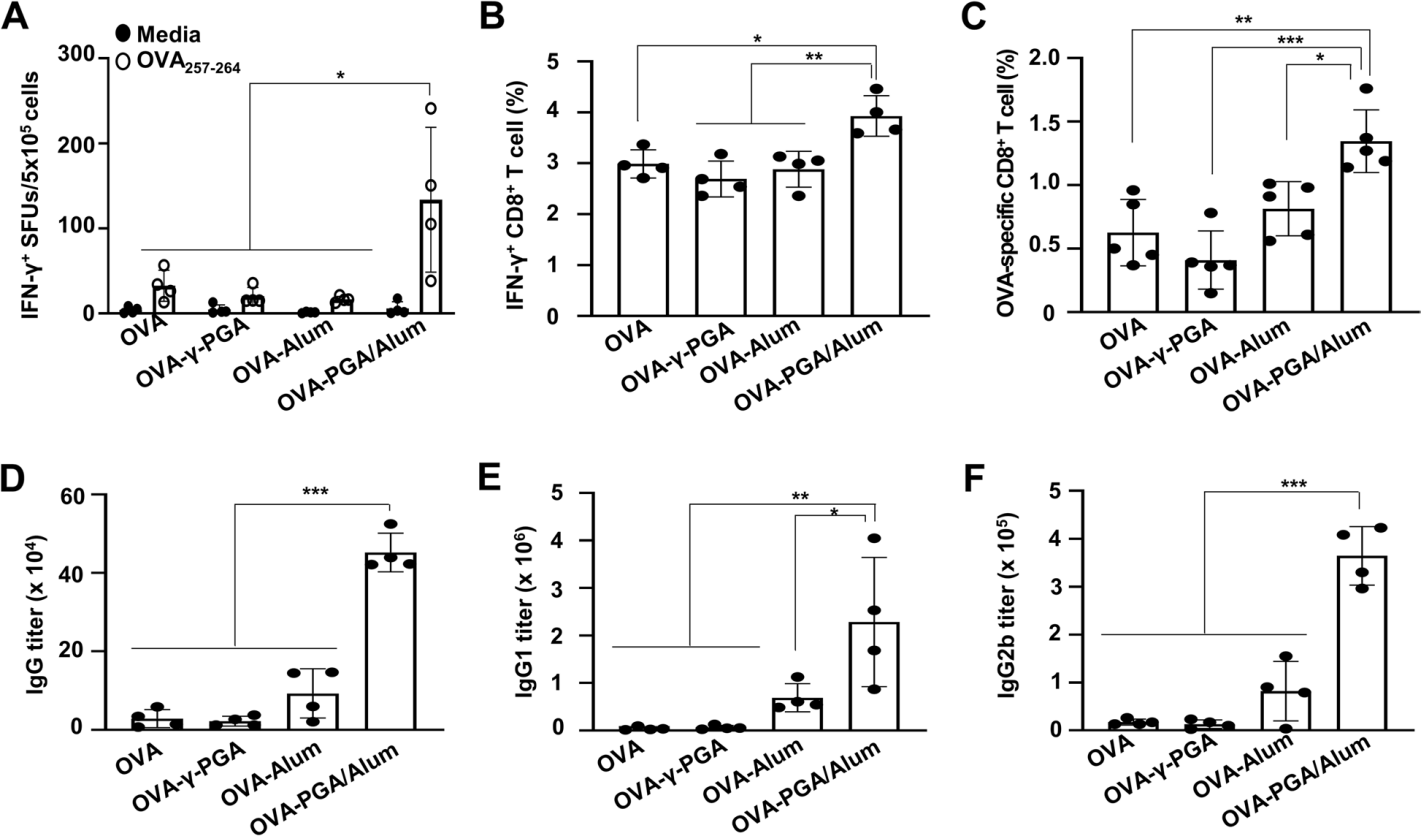
PGA/Alum-adjuvanted OVA-immunized aged mice showed increases in OVA-specific CD8^+^ T cell activation and IgG production. Aged mice (*n* = 4–5 per group) were i.m. administered OVA protein alone or combined with γ-PGA, alum, or PGA/Alum on days 0, 14, and 21. Seven days after the last immunization, the splenocytes and sera were obtained from the mice. **A** IFN-γ^+^ SFUs were enumerated via ELISPOT assays after stimulating splenocytes with OVA_257–264_ for 60 h. **B** Flow cytometry was performed by stimulating splenocytes with OVA_257–264_ in the presence of monensin for 12 h, followed by staining with a fluorescent dye-conjugated anti-CD3ε and anti-CD8α Abs and by further intracellular staining with an anti-IFN-γ-PE Ab. **C** The splenocytes were stained with a fluorescent dye-conjugated anti-CD8α Ab and H-2K^b^-OVA_257–264_ tetramer and then analyzed within CD8^+^ T cell via flow cytometry. **D–F** ELISA was performed using sera to determine Ab titers of OVA-specific IgG (**D**), IgG1 (**E**), and IgG2b (**F**). Statistical significance was analyzed using one-way ANOVA/Bonferroni; *P < 0.05, **P < 0.01, and ***P < 0.001

Since adaptive immune responses are essential for inducing vaccine efficacy [28], PGA/Alum was investigated to determine whether it improves the antigen-specific cellular and humoral immunity in the aged mice (*n* = 4 per group) by i.m. administering OVA alone or in combination with γ-PGA (OVA- γ-PGA), alum (OVA-Alum), or PGA/Alum (OVA-PGA/Alum) on days 0, 14, and 21. Seven days after the last immunization, splenocytes were stimulated with the MHC class I-restricted OVA_257–264_ peptide and subjected to the ELISPOT assay to evaluate the CD8^+^ CTL activity. As shown in Fig. 2A, significantly higher IFN-γ^+^ spot-forming units (SFUs) were observed in the OVA-PGA/Alum group (133 ± 74 SFUs) than in the OVA (33 ± 16 SFUs), OVA-γ-PGA (20 ± 9 SFUs), and OVA-Alum (14 ± 6 SFUs) groups (*P* < 0.05). To clarify clearly the adjuvanticity of PGA/Alum in aged and young mice, we determined the number of the IFN-γ^+^ SFUs in OVA-PGA/Alum immunized aged and young mice and found that the degree of IFN-γ^+^ SFUs was similar between OVA-PGA/Alum-immunized aged and OVA-PGA/Alum-immunized young mice (Fig. S8A). Flow cytometry also showed that the percentages of the IFN-γ^+^ CD8^+^ T cells were significantly higher in the OVA-PGA/Alum-aged mice (3.9 ± 0.3%) than in the OVA- (2.9 ± 0.2%), OVA-γ-PGA- (2.6 ± 0.3%), and OVA-Alum- (2.8 ± 0.3%) aged mice (Fig. 2B and Fig. S9A). Moreover, the generation of OVA_257–264_ tetramer^+^ CD8^+^ T cells was higher in the OVA-PGA/Alum-aged mice (1.3 ± 0.2%) than in the aged mice immunized with OVA (0.6 ± 0.2%), OVA-γ-PGA (0.4 ± 0.2%), and OVA-Alum (0.8 ± 0.2%) (Fig. 2C and Fig. S9B), suggesting that the CTL activity in aged mice could be raised, similar to that of young mice, by PGA/Alum.

Next, the humoral immune response was confirmed by measuring the OVA-specific antibody (Ab) titer in the sera via ELISA. The OVA-specific IgG titers were almost eightfold higher in the OVA-PGA/Alum-aged mice (452,018 ± 49,238 titer) than in the aged mice immunized with OVA (28,594 ± 23,021 titer), OVA-γ-PGA (22,215 ± 12,539 titer), and OVA-Alum (92,935 ± 62,881 titer) (*P* < 0.001) (Fig. 2D). Titers of OVA-specific IgG subclasses, Th1-biased Ab (IgG2b) and Th2-biased-Ab (IgG1), also showed significant twofold increases in the OVA-PGA/Alum-aged mice than in the other aged mice (Fig. 2E and F). We further compared the serum IgG titers in the aged and young mice immunized with OVA-PGA/Alum. Unlike the CTL activity, IgG titers were still tenfold lower in the OVA-PGA/Alum-aged mice than in the OVA-PGA/Alum-young mice (Fig. S8B). The IgG2b titer was also fivefold lower in the OVA-PGA/Alum-aged mice than in the OVA-PGA/Alum-young mice, whereas the IgG1 titer was not different between the groups (Fig. S8C). These results suggest that the use of PGA/Alum in aged mice can improve the antigen-specific CTL activity more effectively than Ab production.

### The use of PGA/Alum as an adjuvant enhances the influenza pH1N1 vaccine antigen-specific immune responses in aged mice

**Fig. 3:**
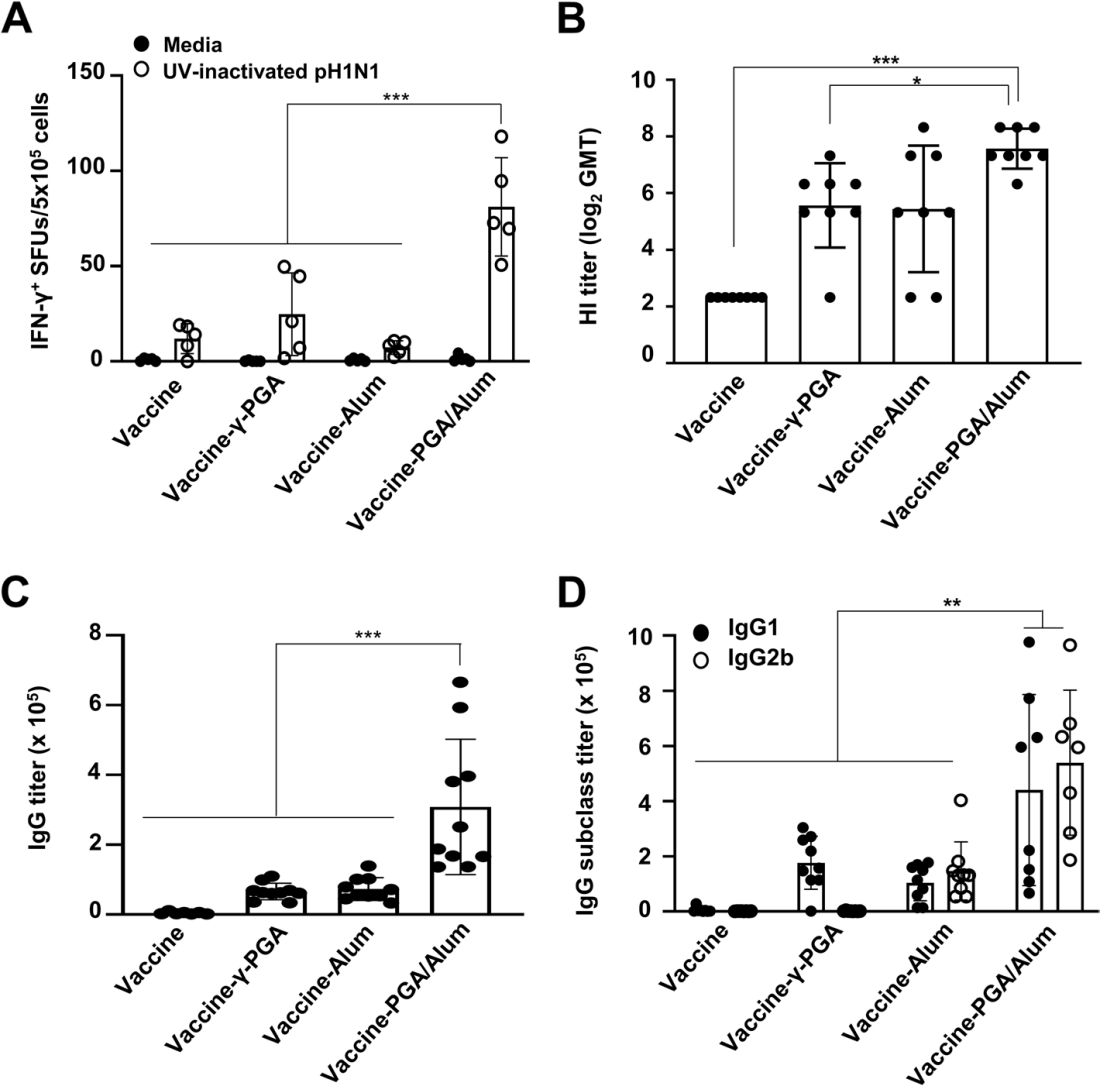
PGA/Alum significantly improves influenza pH1N1 vaccine antigen-specific cell-mediated and humoral immune responses in aged mice. Aged mice (*n* = 5–10 per group) were i.m. administered the pH1N1 split vaccine antigen alone or mixed with γ-PGA, alum, or PGA/Alum on days 0 and 14. The splenocytes and sera were collected 2 weeks after the last immunization. **A** The splenocytes were stimulated with 500 TCID_50_ UV-inactivated pH1N1 for 60 h, and IFN-γ^+^ SFUs were detected via ELISPOT assays. **B** Serum HI titers were measured against the pH1N1 using the sera and are shown as GMT. (**C** and **D)** ELISA was performed to determine sera Ab titers of vaccine antigen-specific IgG (**C**), IgG1, and IgG2b (**D**). Statistical significance was analyzed using one-way ANOVA/Bonferroni; *P < 0.05, **P < 0.01, and ***P < 0.001

To investigate whether the recovery of age-driven immune suppression by PGA/Alum can act similar to that of the commercially available vaccine antigens, the aged mice (*n* = 5–10 per group) were immunized twice with the influenza pH1N1 split-vaccine antigen alone (vaccine) or mixed with γ-PGA (vaccine-γ-PGA), alum (vaccine-Alum), or PGA/Alum (vaccine-PGA/Alum) at 2-week intervals. Two weeks after the last vaccination, the vaccine-specific adaptive immune responses were analyzed using the splenocytes and sera via the IFN-γ ELISPOT assay, hemagglutinin-inhibition, and IgG-specific ELISA assays. The ELISPOT assay was performed by stimulating the splenocytes with a UV-inactivated pH1N1 and revealed that IFN-γ^+^ SFUs were significantly more than threefold higher in the vaccine-PGA/Alum group (81 ± 23 SFUs) compared to the vaccine (11 ± 7 SFUs), vaccine-γ-PGA (24 ± 19 SFUs), and vaccine-Alum (7 ± 3 SFUs) groups (*P* < 0.001) (Fig. 3A). The HI titers against the pH1N1, an indicator of the protective efficacy of the influenza vaccine, were also increased in the sera from the vaccine-PGA/Alum group [190 geometric mean titer (GMT)] compared to those from the vaccine (5 GMT; *P* < 0.001), vaccine-γ-PGA (48 GMT; *P* < 0.05), and vaccine-Alum (44 GMT) groups (Fig. 3B). In addition, a significantly higher pH1N1-specific IgG titer was observed in the sera from the vaccine-PGA/Alum group (308,201 ± 193,963 titer) than in the vaccine (4,321 ± 3,458 titer), vaccine-γ-PGA (66,197 ± 23,618 titer), and vaccine-Alum (73,167 ± 32,633 titer) groups (*P* < 0.001) (Fig. 3C). The Ab titers of IgG subclasses, IgG2b and IgG1, were also significantly higher in the sera from the vaccine-PGA/Alum group than in the other groups (*P* < 0.01) (Fig. 3D). These findings demonstrate that PGA/Alum triggers influenza antigen-specific cellular immune responses in the aged mice, accompanied by an increases in humoral immune responses.

### PGA/Alum suppresses the proportion of age-associated CD8^+^ T cell subset and gene expression of the inhibitory regulators within the CD8^+^ T cells, contributing to a decrease in the dysfunctional CD8^+^ T cells

**Fig. 4:**
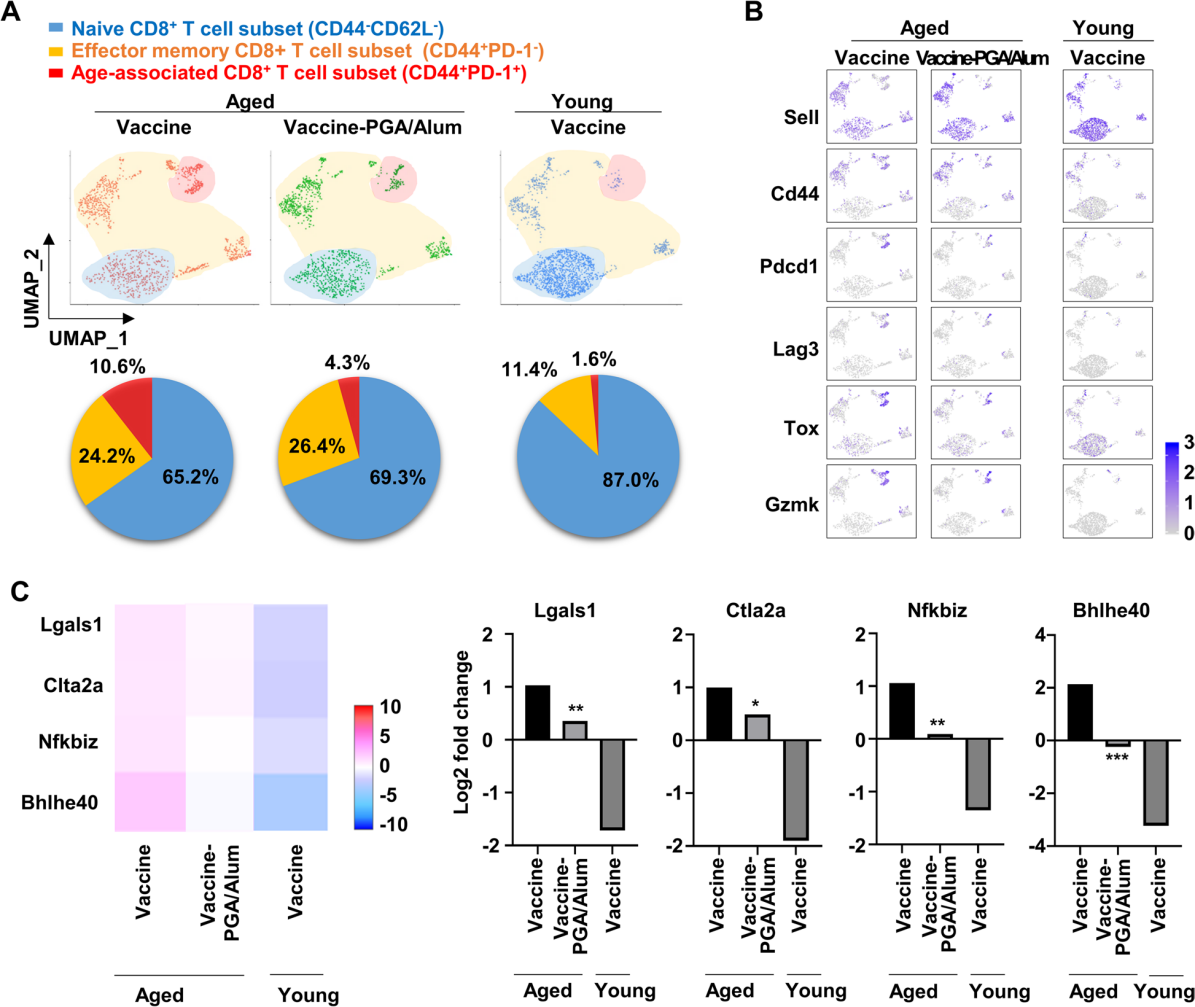
Changes in proportion and gene levels of CD8^+^ T cells in aged mice by PGA/Alum. **A** UMAP dimensionality reduction embedding CD8^+^ T cells within CD45^+^ cells isolated from the splenocytes of the vaccine-aged (1,322 cells), vaccine-PGA/Alum-aged (1,254 cells), and vaccine-young mice (1,793 cells) (pool of *n* = 3 per group) and differential representation of CD8^+^ T cell subsets. **B** Scaled gene expression within CD8^+^ T cells. **C** Heat-map analysis using gene expression profiles within CD8^+^ T cells. The representative significant difference of gene expression with bar graph. *P < 0.05, **P < 0.01, and ***P < 0.001

To analyze the alteration of the immune profile by PGA/Alum in the aged mice in detail, the single-cell RNA analysis, a widely used tool to identify differentially expressed genes within one cell was employed. The scRNA-seq of the immune cells was analyzed using the CD45^+^ cells sorted from the splenocytes of the vaccine-aged mice and vaccine-PGA/Alum-aged mice (pool of *n* = 3 per group) on day 14 after the last immunization. To compare the cellular landscape, the CD45^+^ cells were sorted from the splenocytes of the vaccine-young mice. Clustering of the data was performed based on the key signature genes and standard surface markers and represented in the uniform manifold approximation and projection (UMAP) dimension reduction (Fig. S10A and B). Similar to previous findings [17, 29], the aged mice exhibited lower proportions of the CD8^+^ and CD4^+^ T cells and a higher proportion of the regulatory T cells (Treg) than the young mice, despite immunization with the vaccine (Fig. S10C). Since the functional CD8^+^ T cell subsets are key players in steering the immune responses to execute viral clearance [28], we focused on the CD8^+^ T cell subsets (naive, effector memory, and age-associated subsets). As shown in Fig. 4A, the vaccine-aged mice exhibited a 6.6-fold higher percentage of age-associated (CD44^+^PD-1^+^, 10.6%), 2.3-fold higher percentage of effector memory (CD44^+^PD-1^−^, 24.2%), and 1.33-fold lower percentage of naive (CD44^−^CD62L^−^, 65.2%) subsets than the vaccine-young mice (CD44^+^PD-1^+^, 1.6%; CD44^+^PD-1^−^, 11.4%; CD44^−^CD62L^−^, 87%), as previously reported [29, 30]. The most striking observation was a 2.4-fold lower percentage of the age-associated subset in the vaccine-PGA/Alum-aged mice (4.3%) than in the vaccine-aged mice (10.6%). However, there was little change in the percentages of effector memory and naive subsets: 1.09-fold and 1.06-fold higher in the vaccine-PGA/Alum-aged mice (26.4% and 69.3%) than in the vaccine-aged mice (24.2% and 65.2%), respectively. Within the age-associated CD8^+^ T cell subset, the expression of the phenotypic and transcriptional markers of senescence or exhaustion, including *Pdcd1*, *Tox*, *Lag3*, and *Gzmk* (encoding PD-1, Tox, Lag3, and Granzyme K proteins) was lower in the vaccine-PGA/Alum-aged mice than in the vaccine-aged mice (Fig. 4B). The gene expression profiles were further analyzed because the inhibitory regulators in modulating the T cell activation are important for controlling the CD8^+^ T cell function. As shown in Fig. 4C, the total CD8^+^ T cells of the vaccine-PGA/Alum-aged mice had significantly lower gene expression of T cell inhibitory molecules (*lgals1*, *ctla2a*, *Nfkbiz*, and *Bhlhe40*, encoding Galectin-1, CTLA-2 alpha, NF-Kappa-B inhibitor ζ, and BHE40 protein) than those of the vaccine-aged mice (*P* < 0.05). The PGA/Alum-altered gene levels were the median values between aged and young mice immunized with vaccine alone. These results demonstrate that PGA/Alum can decrease the proportion of age-associated CD8^+^ T cell subsets and gene levels of negative regulators in total CD8^+^ T cell response, consequently resulting in an increase of functional CD8^+^ T cell subset and a decrease of dysfunctional CD8^+^ T cell subset in the aged mice.

### Aged mice immunized with PGA/Alum-adjuvanted influenza vaccine are robustly protected against the influenza virus infection

**Fig. 5:**
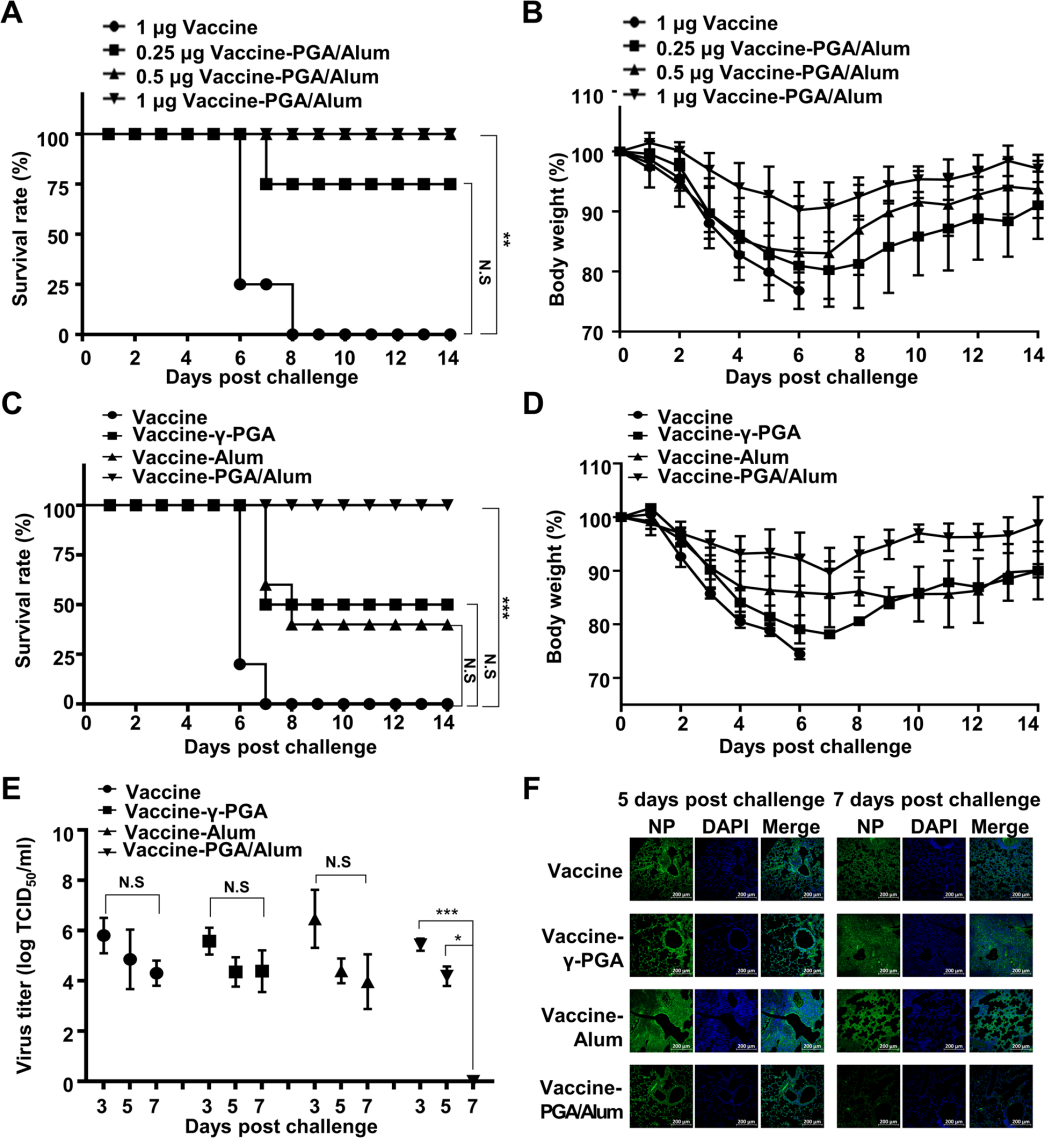
PGA/Alum-adjuvanted pH1N1 vaccine-immunized aged mice drastically protect against pH1N1 virus infection. **A–D** Aged mice (*n* = 5 per group) were i.m. administered the pH1N1 split vaccine alone (0.25, 0.5, or 1 µg) or mixed with PGA/Alum (**A** and **B**) or 0.5 μg the vaccine antigen alone or mixed with γ-PGA, alum, or PGA/Alum (**C** and **D**) on days 0 and 14. Two weeks after the final administration, the mice were i.n. infected with 50 LD_50_ pH1N1 viruses. Survival rates (**A** and **C**) and body weight changes (**B** and **D**) were monitored daily for 14 days post-challenge. **E** Viral titers were measured using lung homogenates (*n* = 3 per group) on days 3, 5, and 7 after the viral challenge and are expressed as log_10_TCID_50_/mL. **F** Five and 7 days post-infection, the left lung was sectioned and stained with anti-influenza nucleoprotein (NP) Ab followed by incubation with Alexa Fluor 488-conjugated anti-mouse IgG. The nuclei were stained with DAPI. Statistical significance was analyzed by log-rank test (**A** and **C**) or by one-way ANOVA/Bonferroni (**E**); *P < 0.05, **P < 0.01, and ***P < 0.001. NS, not-significant

Finally, to evaluate the adjuvanticity of PGA/Alum in the flu vaccine in aged mice, we first immunized young and aged mice (*n* = 5 per group) with the flu vaccine and compared the protection to the pH1N1 virus (A/California/04/09) infection. Similar to the previous reports showing that the flu vaccine efficacy decreases with advancing aging [31, 32], all vaccine-aged mice had died 6 days after the virus challenge (0% survival), whereas all vaccine-young mice survived even though the bodyweight loss occurred (100% survival) (Fig. S11A and B). Next, the aged mice (*n* = 5 per group) were i.m. administered various doses of the pH1N1 vaccine antigen (0.25, 0.5, and 1 μg) alone or mixed with PGA/Alum. As shown in Fig. 5A and B, the vaccine-PGA/Alum group at the highest vaccine dose (1 μg) exhibited a 100% survival rate with very little body weight loss. The vaccine-PGA/Alum group at 0.5 μg of vaccine dose also showed 100% survival despite body weight loss, whereas the group at the lowest vaccine dose (0.25 μg) was partially protected (75% survival). These findings indicate that PGA/Alum can recover the age-driven decline in the protective vaccine efficacy to a similar extent as the efficacy of the vaccine alone in young mice. To elucidate the ability of PGA/Alum as a vaccine adjuvant, the aged mice (*n* = 5 per group) were administered the flu vaccine alone (0.5 μg) or mixed with γ-PGA, alum, or PGA/Alum, and then challenged with the pH1N1 as described above. As expected, the vaccine-PGA/Alum group showed a 100% survival rate, whereas vaccine-γ-PGA group and vaccine-Alum group showed 50% and 40% survival rate, respectively (Fig. 5C and D). All vaccine alone group had died (0% survival, *P* < 0.001).

Since clearance of virus from the lung is a pivotal factor in protective vaccine efficacy, the viral titers were determined in the lung from the vaccinated aged mice (*n* = 3 per group) on days 3, 5, and 7 after the pH1N1 viral challenge. The vaccine-PGA/Alum group was significantly better at clearing the infected virus (Fig. 5E). On day 7 post-challenge, the vaccine-PGA/Alum group exhibited complete viral clearance (*P* < 0.05), whereas the other groups still had high viral loads by day 7. Moreover, the immunofluorescence staining revealed that the presence of influenza virus was very low in the lung section from the vaccine-PGA/Alum group 7 days post-challenge, whereas the virus was strongly detected in the other groups (Fig. 5F). Collectively, these results indicate that the use of PGA/Alum as an adjuvant improves the protection against the influenza virus by facilitating the viral clearance in the aged mice.

## Discussion

Vaccination is the best strategy to control infectious viral diseases, but the poor vaccine efficacy observed in the elderly population remains an inescapable issue demanding a solution [33]. Consistent with previous reports [10, 12], there were lower abilities for antigen trafficking and migration of the antigen-loaded DCs to dLNs, DC activation, and DC-mediated T cell activation in the aged mice than in the young mice. Attempts to develop a good adjuvant to activate DCs in the immunocompromised populations have been made since DC activation is essential to initiate adaptive immunity leading to vaccine efficacy [9]. Adjuvants are primarily used as robust stimulators of innate immune responses, like TLR agonists [31, 34]. γ-PGA is an edible safe biopolymer capable of TLR4-mediated DC activation and subsequent elicitation of adaptive immunity [35]. Alum is an approved adjuvant in humans and increases the antigen persistency at the injection site leading to enhancing Ab production but does not trigger CTL responses. Based on our previous report showing that PGA/Alum did not affect cell viability of bone marrow-derived DCs in vitro and showed no adverse effects including change of body temperature and severe systemic inflammation in young mice in vivo [24], we speculated that PGA/Alum could be a safe adjuvant in aged mice. To reinforce the adjuvanticity of γ-PGA, a PGA/Alum complex was fabricated. Indeed, PGA/Alum had more robust adjuvanticity than γ-PGA or alum only, with significant increases in DC activation and migration and the vaccine-specific adaptive immunities in the young mice [24], implying the possibility of PGA/Alum as an adjuvant for the elderly. In particular, we revealed that the PGA/Alum-recovered functions of DCs from the aged mice were similar to those of the DCs from young mice.

We determined whether the impaired adaptive immune responses in the aged mice were recovered by PGA/Alum and revealed that PGA/Alum robustly increased the OVA-specific IFN-γ production by CD8^+^ T cells in the aged mice, to a similar extent as that of the young mice. Indeed, the PGA/Alum-adjuvanted flu vaccine-immunized aged mice showed a robust increase in virus-specific IFN-γ secreting T cells compared to the vaccine alone-immunized aged mice. As the recent studies reported [17, 27, 30], our results also showed that the age-associated dysfunctional CD8^+^ T cells were rarely found in young mice (1.6%), but its proportion was increased in aged mice (10.6%). Intriguingly, in aged mice, PGA/Alum decreased proportion of the age-associated dysfunctional CD8^+^ T cells by nearly half (4.3%) (Fig. 4A). Currently, several studies have suggested that DC-mediated CD40 activation induce the decrease of dysfunctional CD8^+^ T cells generated in animal models mimicking chronic viral infection [36–38]. CD40 is one of co-stimulatory molecules on DCs responsible for initiating T cell activation, and the CD40 activation using agonistic anti-CD40 Ab was reported to decrease proportion of dysfunctional CD8^+^ T cells [36]. Another study also showed that the CD40 signaling reverts dysfunctional CD8^+^ T cells via mTORC1 pathway [37, 38]. Based on our results showing that PGA/Alum increased expression of CD40 on DCs from aged mice (Fig. 1D), we speculate that PGA/Alum-mediated CD40 activation may be attributed to the decreased proportion of age-associated CD8^+^ T cells in aged mice. Additionally, we analyzed the proportion of CD4^+^ T cell subsets, but no change was observed in the aged mice immunized with vaccine alone and vaccine-PGA/Alum (Fig. S12).

With aging, CD8^+^ T cells become more dysfunctional or anergic cells resembling exhausted CD8^+^ T cells with high levels of negative regulators, and the age-associated T cell subset emerges uniquely, consequently hindering effective protective immunity against the pathogens [39]. In aspect of the vaccine efficacy, the increase of functional CD8^+^ T cells is important to protect against influenza virus infection due to its function to eliminate infected cells [28]. The age-associated dysfunctional CD8^+^ T cells have reported to express various inhibitory molecules (PD-1, LAG3, and TOX3) [17, 29], which suppress functional CD8^+^ T cell activation thereby leading to the reduced vaccine efficacy [40]. Our results demonstrated that PGA/Alum decreased proportion of the age-associated CD8^+^ T cells along with reduction in expression of inhibitory molecules in aged mice (Fig. 4B and C). Thus we speculate that PGA/Alum-mediated decrease of the age-associated CD8^+^ T cells in aged mice may improve the CD8^+^ T cell-mediated vaccine efficacy. Further studies are required to understand the immunological mechanism of the age-associated dysfunctional CD8^+^ T cell subset.

Finally, we evaluated the adjuvant effect of PGA/Alum on the protective efficacy of the flu vaccine after the virus challenge in the aged mice. The PGA/Alum-adjuvanted flu vaccine induced 100% survival of the aged mice, whereas all flu vaccine alone-immunized aged mice had died. The PGA/Alum-recovered vaccine efficacy in the aged mice was accompanied by increases in the virus-specific CTL activity, HI titers, IgG titers, and viral clearance following homologous viral challenge. CD8^+^ T cells have cross-reactivity with other subtypes of influenza viruses [41, 42]; we have also previously demonstrated the PGA/Alum-enhanced cross-protection of the flu vaccine in the young mice [24]. Further studies are warranted to determine the adjuvant effect of PGA/Alum on the heterosubtypic cross-reactivity of the vaccine in aged mice.

Currently, MF59 is the only approved adjuvant for the flu vaccine for the elderly [22]. Clinical studies have reported that the MF59-adjuvanted flu vaccine enhances the vaccine efficacy in the elderly, with increasing Ab titers, neutralizing responses, and T cell activation [22, 43]. However, the underlying mechanisms of adjuvants in the recovery of the age-driven decline in vaccine efficacy remain poorly understood. To our knowledge, this is the first report to reveal a decrease in the population of age-associated CD8^+^ T cell subset and levels of negative regulators of the CD8^+^ T cell response by the adjuvant. Coronavirus disease 19 (COVID-19) caused by severe acute respiratory syndrome-2 (SARS-CoV-2) virus infection has emerged as a global pandemic and is more susceptible to the elderly [44]. Similar to influenza viral infections, the innate and adaptive immune responses play an important role in SARS-CoV-2 infection [45]. Further investigations are warranted to determine whether PGA/Alum acts as a potent vaccine adjuvant to benefit human clinical trials involving the elderly population.

## Conclusions

Here, we revealed that PGA/Alum ameliorates the age-driven reduction of antigen trafficking, DC migration and activation, DC-mediated CD8^+^ T cell activation, thereby leading to CTL activity. Notably, the degree of PGA/Alum-increased IFN-γ^+^ CD8^+^ T cells in aged mice was similar to those in young mice. Importantly, PGA/Alum decreased proportion of age-associated CD8^+^ T cell subset and gene expression of inhibitory regulators in CD8^+^ T cells, contributing to an increase of functional CD8^+^ T cells in aged mice. Overall, the use of PGA/Alum in flu vaccine conferred full protection against influenza virus infection in aged mice while all aged mice immunized with vaccine alone died. Taken together, these results suggest that PGA/Alum could be a potent vaccine adjuvant that improves the reduced vaccine efficacy of aging by recovering DC functions and CD8^+^ T cell activity, thereby leading to the prevention of influenza virus infection in the elderly.

## Methods

### Mice and cells

Eighteen-month-old (aged) female C57BL/6 mice were maintained at the Laboratory Animal Resource Center of the Korea Research Institute of Bioscience and Biotechnology (KRIBB). Six-week-old female C57BL/6 mice and OT-I transgenic mice were purchased from OrientBio (Gyeonggido, Republic of Korea) and Jackson Laboratory (ME, USA), respectively. All animal experiments were approved by the Institutional Animal Care and Use Committee (IACUC) of KRIBB (Approval number: KRIBB-AEC-20042) and performed under the guidelines in a specific pathogen-free facility. Splenocytes were cultured in the RPMI 1640 medium with 10% heat-inactivated fetal bovine serum (FBS), 100 U/mL penicillin, and 100 mg/mL streptomycin (Gibco, NY, USA). Splenic DCs were isolated using mouse CD11c MicroBeads UltraPure (Miltenyi Biotec, Bergisch Gladbach, Germany), and the purity was > 80%. Madin-Darby canine kidney (MDCK) cells (ATCC, VA, USA) were maintained in EMEM medium (Lonza, MD, USA) supplemented with 5% heat-inactivated FBS, 1% MEM vitamin solution (Sigma-Aldrich Chemical Co., MO, USA), 100 U/mL penicillin, and 100 mg/mL streptomycin (Gibco).

### Adjuvants and antigens

PGA/Alum was fabricated by combining γ-PGA (BioLeaders, Daejeon, Republic of Korea) and Imject alum (Thermo Fisher, MA, USA) as described previously [24]. OVA protein and pH1N1 split vaccine antigen (A/California/7/2009 NYMC X-179A H1N1) were obtained from Sigma-Aldrich and Mogam Biotechnology Research Institute (Gyeonggido, Republic of Korea), respectively.

### Virus preparation

The influenza virus A/California/4/2009 (pH1N1) was proliferated in the allantoic cavities of 10-day-old SPF embryonated chicken eggs, harvested from the allantoic fluid, centrifuged at 2,580 × *g* for 20 min, and then stored at -80 °C until use. All experiments were performed under biosafety level 2 setting.

### Mouse immunization and viral challenge

Mice were i.m. administered 10 μg OVA protein alone or combined with 400 μg γ-PGA, 400 μg alum, or 800 μg PGA/Alum on days 0, 14, and 21. In a separate experiment, mice were i.m. administered the pH1N1 vaccine containing 0.5 μg HA alone or combined with 400 μg γ-PGA, 400 μg alum, or 800 μg PGA/Alum on days 0 and 14. The sera and splenocytes were collected on day 7 or 10 after the last immunization. In other experiments, mice were i.m. administered the pH1N1 vaccine containing 0.25, 0.5, or 1 μg hemagglutinin (HA) combined with 800 μg PGA/Alum on days 0 and 14. Fourteen days after the final vaccination, the mice were challenged intranasally (i.n.) with 50 LD_50_ of pH1N1 virus, and survival rate and body weight were monitored for up to 14 days. Mice that lost more than 25% of their body weight reached the experimental endpoint.

### In vivo fluorescence imaging

For in vivo visualization of antigen trafficking, IR800-labeled OVA was prepared and administered as previously described [24]. The in vivo NIR fluorescence from the anesthetized mice was acquired using an in vivo imaging system (IVIS Lumina II; Xenogen Corp.) with excitation and emission wavelengths of 780 and 831 nm, respectively. The MFIs of OVA-IR800 in the axillary LNs were quantitatively analyzed using ImageJ software (NIH, MD, USA).

### Flow cytometry

Cells were incubated with anti-CD16/32Ab (BD Bioscience, CA, USA) to block Fc receptors for 15 min at 4 °C before staining. To test the DC migration, the mice were i.m. injected with 5 µg Alexa Fluor 647-conjugated OVA (Thermo Fisher) alone or mixed with 400 µg alum, 400 µg γ-PGA, or 800 µg PGA/Alum. At 3 h post-immunization, the muscle in injected site and iliac LNs were obtained and treated with DNase I and liberase (Sigma-Aldrich) for 30 min at 37 °C. After removing debris using a 70 µm nylon cell strainer (BD, NJ, USA), the cells were washed with 1% FBS containing PBS once, blocked with Fc receptors, and stained with anti-CD11c-PE and anti-CD3ε-PerCP Abs. MFIs in DCs were analyzed within CD11c^+^CD3^−^ population. To analyze DC activation, splenic DCs were stimulated with 100 μg/mL γ-PGA, 100 μg/mL alum, and 200 μg/mL PGA/Alum for 30 h at 37 °C, followed by staining with anti-CD11c-APC efluor780, anti-CD40-PE, and anti-CD86-PerCP Abs. To assess DC-mediated T cell activation, the splenic DCs were stimulated as described above for 6 h at 37 °C, washed, and then co-cultured with OT-I CD8^+^ T cells isolated from splenocytes of OT-I mice using a CD8α^+^ T cell isolation kit (Miltenyi Biotec), for 6 h at 37 °C in the presence of monensin (BD Bioscience). For T cell proliferation, the treated DCs were mixed with OT-I CD8^+^ T cells labeled using a CellTrace CFSE cell proliferation kit (Thermo Fisher) according to the manufacturer’s instructions for 5 days at 37 °C. The mixed cells were stained with anti-CD3ε-PerCP-Cy5.5 and anti-CD8α-APC Abs. Intracellular staining was performed using anti-IFN-γ-PE Ab. To determine the percentage of OVA-specific IFN-γ^+^ T cells, splenocytes were stimulated with 5 μg/mL OVA_257–264_ in the presence of monensin for 12 h at 37 °C and then stained with anti-CD3ε-PerCP-Cy5.5 and anti-CD8α-FITC Abs, followed by intracellular staining with anti-IFN-γ-PE Ab. The CD8^+^ T cells were analyzed in the CD3^+^CD8^+^ population. Generation of the OVA-specific CD8^+^ T cells was determined by staining with H-2K^b^ OVA_257–264_ tetramer-APC and anti-CD8α-FITC Ab (MBL International Corporation, MA, USA). All the Abs were purchased from BD Bioscience, BioLegend (CA, USA), eBioscience (CA, USA), or Thermo Fisher. The stained cells were acquired using a FACSverse (BD Bioscience) flow cytometer and analyzed using FlowJo software (Tree Star, CA, USA).

### ELISPOT assay

The frequency of IFN-γ-producing cells was evaluated using mouse IFN-γ ELISPOT kits (BD Bioscience). Briefly, the splenocytes were plated at 5 × 10^5^ cells/well onto purified IFN-γ Ab-coated ELISPOT plates and stimulated with 1 μg/mL OVA_257–264_ or 500 TCID_50_/mL of UV-inactivated pH1N1 virus for 60 h at 37 °C. The SFUs were enumerated using an ELISPOT plate reader (Cellular Technology Ltd., OH, USA).

### ELISA

For measurement of the levels of antigen-specific Ab, ELISA plates were coated with 0.5 μg/mL OVA protein or 0.5 μg/mL pH1N1 split vaccine, washed with PBS 3 times, and blocked with 5% skim milk in PBST. The sera was reacted to the antigen-coated plates followed by incubation with the HRP-anti-mouse IgG (Cell Signaling, MA, USA), IgG1, or IgG2b (SouthernBiotech, AL, USA). The plates were washed and developed with the chromogenic tetramethylbenzidine substrate (BD Bioscience) and the reactions were terminated with 2 N H_2_SO_4_. The absorbance was measured at 450 nm using a Versamax microplate reader (Molecular Devices, CA, USA).

### Single-cell RNA-seq and data processing

Splenocytes were filtered through a 70 μm filter to remove debris and incubated with anti-mouse CD16/32 Ab followed by staining with anti-CD45 Ab-APC and live/dead fixable red dead cell stain (Thermo Fisher) for 30 min. The live CD45^+^ cells were sorted using a FACS Aria Fusion (BD Biosciences), and the cell pellet was resuspended in 0.04% BSA-PBS at 1,000 cells/μL. The single-cell RNA-seq libraries were generated using the Chromium Next gel beads-in-emulsion (GEM) single-cell 5 Kit v2 (10 × Genomics, CA, USA) at the BioMedical Research Center, Korea Advanced Institute of Science and Technology (Daejeon, Republic of Korea) following the manufacturer’s instructions. The libraries were sequenced at a depth of approximately 40,000 reads per cell using the Novaseq 6000 platform (Illumina, CA, USA) (300 cycles) by Macrogen (Seoul, Republic of Korea).

Sample demultiplexing, barcode processing, and single-cell 5′ counting was performed using Cell Ranger 5.0.0 (10 × Genomics). The cell ranger cell count was used to align samples to the reference genome (mm10 for mouse genome; mm10-2020-A, GRCm38-alts-ensembl-5.0.0), quantify reads, and filter reads with a quality score below 30. The Seurat 3.1.3 R package was used for cell population analysis. Cells with mitochondrial ratios over 5% and unique feature count over 5,000 (doublets or multiplets) less than 200 (low-quality cells or empty droplets) were removed (Fig. S13). The filtered data were normalized using a scaling factor of 10,000 and the final filtered data were a feature-barcode matrix with 18,506 genes and 30,602 cells from the spleen.

For clustering cells, a K-nearest neighbor (KNN) graph was constructed based on the Euclidean distance in PCA space, and the edge weights between any two cells were refined based on the shared overlap in their local neighborhoods (Jaccard similarity), followed by applying modularity optimization techniques such as the Louvain algorithm (default) to iteratively group cells together, to optimize the standard modularity function. The datasets are projected as UMAP plots.

To identify biomarker-defining clusters, we compared a single cluster with all the remaining clusters using the Wilcoxon rank-sum test. The following parameters were used: min.pct = 0.25, logfc.threshold = 0.25, and only the positive ones were filtered.

### Hemagglutination-inhibition (HI) assay

For titration of neutralizing Abs, the sera were treated with the receptor destroying enzyme (Denka Seiken, Tokyo, Japan) for 18 h at 37 °C, and heat-inactivated for 30 min at 56 °C. The serially twofold diluted sera were treated with 4-HA units of pH1N1 for 30 min at 37 °C. Then, turkey red blood cells (tRBCs; 0.7% in PBS) were added and incubated for 30 min at 25 °C to react hemagglutination. The HI titers were calculated by determining the highest dilution factor of each serum that inhibited the hemagglutination of tRBCs.

### Lung virus titration

For determining viral titers in the infected lung, the lungs were harvested and frozen at -80 ℃ at 3, 5, and 7 days post-infection. The homogenates were prepared from the frozen tissues by homogenization with MEM media by TissueLyser II (Qiagen, Venlo, Netherlands) and centrifugation at 15,000 × *g* for 10 min. The MDCK cells were treated with serially tenfold diluted homogenates for 1 h at 37 ℃. The homogenates were removed and incubated with MEM media plus 1% antibiotics, 1% vitamin, 0.2% BSA, and 0.5 μg trypsin-TPCK for 72 h at 37 ℃. The supernatants were mixed with tRBCs for 30 min at 25 ℃, and the viral titers were calculated by the Reed—Muench method and expressed as 50% tissue culture infective dose (TCID_50_).

### Immunofluorescence analysis

For detection of the virus in the infected lung, the inferior lobes of the lung were excised 5 or 7 days post-infection and fixed in 3.7% formaldehyde. The paraffin-embedded tissue sections were prepared and stained with anti-influenza A nucleoprotein Ab (SouthernBiotech), followed by incubation with Alexa Fluor 488-anti-mouse IgG Ab (Thermo Fisher). The nuclei were counterstained with 4′6-diamidino-2-phenylindole (DAPI; Molecular Probes). The tissue sections were observed using a Zeiss LSM 700 confocal laser scanning microscope with ZEN software (Carl Zeiss GmbH, Jena, Germany).

### Statistical analysis

All experiments were performed at least three times, and the statistical significance of differences was evaluated using Student’s two-tailed *t*-test or one-way ANOVA followed by Bonferroni’s correction (ANOVA/Bonferroni). The log-rank test was used to analyze the survival between the two groups. All analyses were performed using the PRISM software (GraphPad Software, Inc., CA, USA), and an asterisk (*) indicates a significant difference between the two groups (*P* < 0.05).

## Supplementary information

**Additional file 1:** **Fig. S1.** Flow cytometry strategies for analyzing recruited DCs and antigen-loaded DCs in injected muscle. Gating strategy of CD11c^+^CD3^-^ cells (recruited DCs) and OVA^+^CD11c^+^CD3^-^ cells (antigen-loaded DCs) and representative dot plots in each group. Numbers in representative dot plots indicate the number of positive cells. **Fig. S2.** Flow cytometry strategies for analyzing total DCs and antigen-loaded DCs in dLNs. Gating strategy of CD11c^+^CD3^-^ DCs and OVA^+^CD11c^+^CD3^-^ DCs and representative dot plots in each group. Numbers in representative dot plots indicate the number of positive cells. **Fig. S3.** PGA/Alum treatment increases the recruitment to injected sites and migration to draining LN of DCs and antigen-loaded DCs from aged mice. Aged (18-month-old) and young (6-week-old) mice (*n* = 3 per group) were i.m. immunized with Alexa647-OVA alone or mixed with γ-PGA, Alum, or PGA/Alum. On 12 h post-injection, the number of total DCs (gated as CD11c^+^CD3^-^ cells) and Fluor-OVA^+^ DCs (gated as Fluor-OVA^+^CD11c^+^CD3^-^ cells) were analyzed in injected muscle region (**A**) and dLNs (**B**) via flow cytometry. Statistical significance was analyzed by one-way ANOVA/Bonferroni; *P < 0.05, **P < 0.01, and ***P < 0.001. **Fig. S4.** Flow cytometry strategy for analyzing expression of co-stimulatory molecules on DCs. Gating strategy of CD40 and CD80 on CD11c^+^ DCs and representative dot plots in each group. Numbers in representative dot plots indicate the percentage of positive cells. **Fig. S5.** PGA/Alum robustly induces the production of inflammatory cytokines in DCs from the aged mice. Splenic DCs were isolated from aged mice and stimulated with 100 μg/mL of γ-PGA, 100 μg/mL of alum, or 200 μg/mL of PGA/Alum for 30 h. Levels of the cytokines were determined in the culture supernatants using a Legendplex immunoassay kit. Statistical significance was analyzed by one-way ANOVA/Bonferroni; *P < 0.05. N.D, not-detected. **Fig. S6.** PGA/Alum increases antigen uptake and processing on DCs from the aged mice. CD11c^+^ DCs were purified from splenocytes of young and aged mice (*n* = 4 per group) and then incubated with FITC-OVA (**A**) or DQ-OVA (**B**) alone or combined with 100 μg/mL of γ-PGA, 100 μg/mL of alum, or 200 μg/mL of PGA/Alum for 6 or 18 h, respectively. Percentages of the FITC-OVA^+^ DCs and DQ-OVA^+^ DCs were analyzed via flow cytometry and the fold-change values were calculated in comparison with FITC-OVA or DQ-OVA alone-treated cells of the aged mice. Statistical significance was analyzed by one-way ANOVA/Bonferroni; *P < 0.05, **P < 0.01, and *** *P *<0.001. **Fig. S7.** Flow cytometry strategies for analyzing CFSE^-^ CD8^+^ T cells and IFN-γ^+^ CD8^+^ T cells in splenocytes. **A** Gating strategy of CFSE-labeled CD8^+^ T cells and representative histograms in each group. Numbers above the bracketed lines in representative histograms indicate the percentage of CFSE^-^ (proliferated) and CFSE^+^ cells. **B** Gating strategy of IFN-γ^+^CD8^+^CD3^+^ T cells and representative dot plots in each group. Numbers in representative dot plots indicate the percentage of positive cells. **Fig. S8.** PGA/Alum enhances antigen-specific cellular immune response of the aged mice as much as the young mice rather than humoral immune response. Young and aged mice (n = 3 per group) were i.m. immunized with OVA alone or mixed with PGA/Alum on days 0, 14, and 21. Seven days after last immunization, the splenocytes and sera were obtained. (**A**) The splenocytes were stimulated with 1 μg/mL of OVA_257-264_ peptide for 60 h, and IFN- γ^+^ SFUs were detected via ELISPOT assays. (**B** and **C**) ELISA was performed to determine antibody titers of OVA-specific IgG (B), IgG1, and IgG2b (C). Statistical significance was analyzed by one-way ANOVA/Bonferroni; *P<0.05, **P<0.01, and ***P<0.001. N.S, not significant. **Fig. S9.** Flow cytometry strategies for analyzing IFN-γ^+^ CD8^+^ T cells and OVA_257-624_ tetramer^+^ CD8^+^ T cells in splenocytes. (**A**) Gating strategy of IFN-γ^+^CD8^+^CD3^+^ T cells and representative dot plots in each group. Numbers in representative dot plots indicate the percentage of positive cells. (**B**) Gating strategy of OVA_257-624_ tetramer^+^ CD8^+^ T cells and representative dot plots in each group. Numbers in representative dot plots indicate the percentage of OVA_257-624_ tetramer^+^ and OVA_257-624_ tetramer^-^ cells. **Fig. S10. **Immune cell landscape of the aged mice immunized with vaccine alone or combined with PGA/Alum. **A** UMAP dimensionality reduction embedding CD45^+^ cells isolated from the splenocytes from aged (pool of *n* = 3, 18-month-old) mice immunized with vaccine (10,042 cells) or vaccine-PGA/Alum (11,212 cells) and vaccine-immunized young (pool of *n* = 3, 6-week-old, 10,042 cells). **B** Violin plots showing scaled expression levels of marker genes per cluster that are used to select clusters for the downstream analysis. **C** The proportions of T cell subsets in the annotated clusters. NK, natural killer. ML NK, memory-like NK. NKT, natural killer T. Mɸ, macrophage. cDC, conventional dendritic cell. pDC, plasmacytoid dendritic cell. **Fig. S11. **None of the aged mice are protected against the pH1N1 virus infection despite immunization of the pH1N1 split vaccine. Young (6-week-old) and aged (18-month-old) mice were i.m. administered the pH1N1 split vaccine antigen on days 0 and 14. **A, B** Two weeks after the final administration, the vaccinated mice were i.n. challenged with 50 LD_50_ pH1N1 virus. Survival rates (**A**) and body weight changes (**B**) were monitored for up to 14 days post-challenge. Each data point represents an average percentage. Statistical significance was analyzed by log-rank test (**A**); ***P<0.001. **Fig. S12.** Effects of PGA/Alum on CD4+ T cell populations in the influenza vaccine-immunized aged mice. **A** UMAP dimensionality reduction embedding CD4^+^ T cells within CD45^+^ cells isolated from the splenocytes from the aged (pool of *n* = 3, 18-month-old) mice immunized with vaccine (1,878 cells) or vaccine-PGA/Alum (1,836 cells), and vaccine-immunized young (pool of n = 3, 6-week-old, 1,793 cells) and differential representation of CD4^+^ T cell subset. **B** Scaled gene expression of CD4^+^ T cells. **Fig. S13.** Quality control for analyzing scRNA-seq and immune profiles. RNA count, number of expressed genes, and percentage of mitochondrial UMI per scRNA-seq sample.

## Data Availability

The data supporting the findings of this study are available from the corresponding author upon reasonable request, without hesitancy, to qualified researcher.

## Abbreviations

Ab: Antibody
BHE40: Basic helix-loop-helix family member E40
CD: Cluster of differentiation
CFSE: Carboxyfluorescein succinimidyl ester
CTLs: Cytotoxic lymphocytes
CTLA-2 alpha: Cytotoxic T lymphocyte antigen-2 alpha
DCs: Dendritic cells
ELISA: Enzyme-linked immunosorbent assay
ELISPOT: Enzyme-linked immunospot
Flu: Influenza
GMT: Geometric mean titer
Gzmk: Granzyme K
HI: Hemagglutination inhibition
IFN-γ: Interferon-γ
IgG: Immunoglobulin G
Lag3: Lymphocyte activating 3
IL-1α: Interleukin-1α
IL-1β: Interleukin-1β
IL-6: Interleukin-6
MCP-1: Monocyte chemoattractant protein-1
MFIs: Mean fluorescence intensities
MHC: Major histocompatibility complex
NF-Kappa-B: Nuclear factor kappa-light-chain-enhancer of activated B cells
NIR: Near-infrared
OVA: Ovalbumin
PD-1: Programmed cell death-1
PGA/Alum: Poly-γ-glutamic acid and alum
PBS: Phosphate-buffered saline
SFUs: Spot-forming units
TLR: Toll-like receptor
TNF-α: Tumor necrosis factor-α
Treg: Regulatory T cells
UMAP: Uniform manifold approximation and projection
dLNs: Draining lymph nodes
i.n.: Intranasally
i.m.: Intramuscularly
pH1N1: Pandemic H1N1
scRNA-seq: Single-cell RNA sequencing
