# Supplementary material for: Poly-γ-glutamic acid/Alum adjuvanted pH1N1 vaccine-immunized aged mice exhibit a significant increase in vaccine efficacy with a decrease in age-associated CD8+ T cell proportion in splenocytes
Source: Immun Ageing. 2022 May 23;19:22. doi: 10.1186/s12979-022-00282-z (PMC9124744; doi:10.1186/s12979-022-00282-z)
Supplement: Supplementary file 1 — Additional file 1: Fig. S1. Flow cytometry strategies for analyzing recruited DCs and antigen-loaded DCs in injected muscle. Gating strategy of CD11c+CD3- cells (recruited DCs) and OVA+CD11c+CD3- cells (antigen-loaded DCs) and representative dot plots in each group. Numbers in representative dot plots indicate the number of positive cells. Fig. S2. Flow cytometry strategies for analyzing total DCs and antigen-loaded DCs in dLNs. Gating strategy of CD11c+CD3- DCs and OVA+CD11c+CD3- DCs and representative dot plots in each group. Numbers in representative dot plots indicate the number of positive cells. Fig. S3. PGA/Alum treatment increases the recruitment to injected sites and migration to draining LN of DCs and antigen-loaded DCs from aged mice. Aged (18-month-old) and young (6-week-old) mice (n = 3 per group) were i.m. immunized with Alexa647-OVA alone or mixed with γ-PGA, Alum, or PGA/Alum. On 12 h post-injection, the number of total DCs (gated as CD11c+CD3- cells) and Fluor-OVA+ DCs (gated as Fluor-OVA+CD11c+CD3- cells) were analyzed in injected muscle region (A) and dLNs (B) via flow cytometry. Statistical significance was analyzed by one-way ANOVA/Bonferroni; *P < 0.05, **P < 0.01, and ***P < 0.001. Fig. S4. Flow cytometry strategy for analyzing expression of co-stimulatory molecules on DCs. Gating strategy of CD40 and CD80 on CD11c+ DCs and representative dot plots in each group. Numbers in representative dot plots indicate the percentage of positive cells. Fig. S5. PGA/Alum robustly induces the production of inflammatory cytokines in DCs from the aged mice. Splenic DCs were isolated from aged mice and stimulated with 100 μg/mL of γ-PGA, 100 μg/mL of alum, or 200 μg/mL of PGA/Alum for 30 h. Levels of the cytokines were determined in the culture supernatants using a Legendplex immunoassay kit. Statistical significance was analyzed by one-way ANOVA/Bonferroni; *P < 0.05. N.D, not-detected. Fig. S6. PGA/Alum increases antigen uptake and processing on DCs from the [file 12979_2022_282_MOESM1_ESM.docx]

**Supporting information**

**Poly-γ-glutamic acid/Alum adjuvanted pH1N1 vaccine-immunized aged mice exhibit a significant increase in vaccine efficacy with a decrease in age-associated CD8^+^ T cell proportion in splenocytes**





**Supplementary Fig. 1** Flow cytometry strategies for analyzing recruited DCs and antigen-loaded DCs in injected muscle. Gating strategy of CD11c^+^CD3^-^ cells (recruited DCs) and OVA^+^CD11c^+^CD3^-^ cells (antigen-loaded DCs) and representative dot plots in each group. Numbers in representative dot plots indicate the number of positive cells.


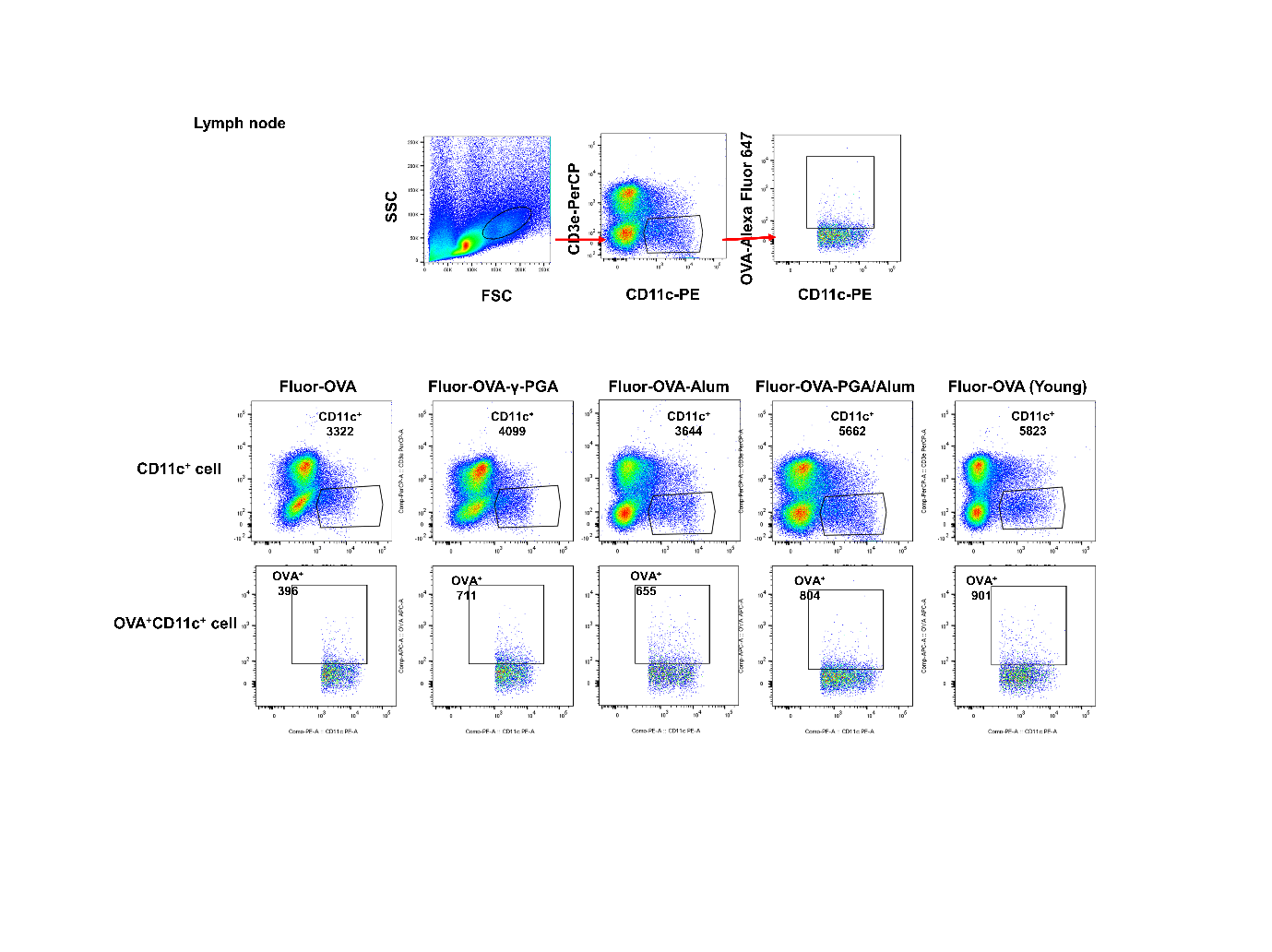


**Supplementary Fig. 2** Flow cytometry strategies for analyzing total DCs and antigen-loaded DCs in dLNs. Gating strategy of CD11c^+^CD3^-^ DCs and OVA^+^CD11c^+^CD3^-^ DCs and representative dot plots in each group. Numbers in representative dot plots indicate the number of positive cells.


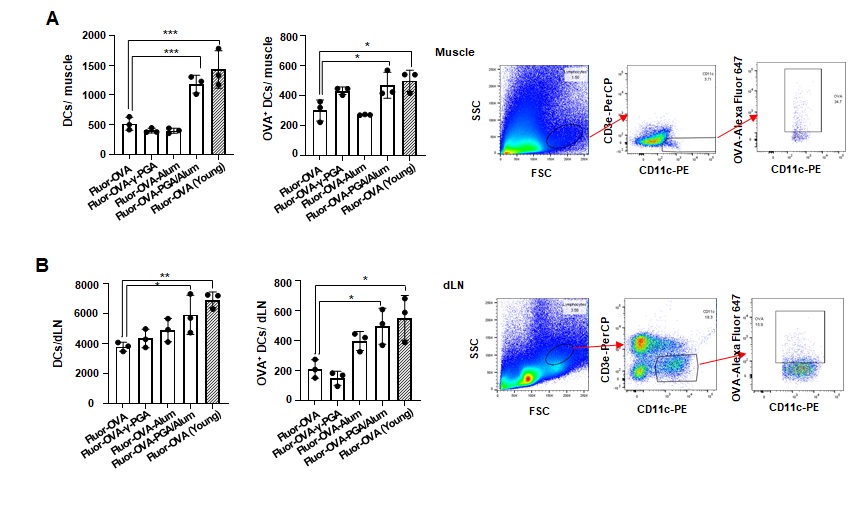


**Supplementary Fig. 3** PGA/Alum treatment increases the recruitment to injected sites and migration to draining LN of DCs and antigen-loaded DCs from aged mice. Aged (18-month-old) and young (6-week-old) mice (n = 3 per group) were i.m. immunized with Alexa647-OVA alone or mixed with γ-PGA, Alum, or PGA/Alum. On 12 h post-injection, the number of total DCs (gated as CD11c^+^CD3^-^ cells) and Fluor-OVA^+^ DCs (gated as Fluor-OVA^+^CD11c^+^CD3^-^ cells) were analyzed in injected muscle region (**A**) and dLNs (**B**) via flow cytometry. Statistical significance was analyzed by one-way ANOVA/Bonferroni; **P* < 0.05, ***P* < 0.01, and ****P* < 0.001.


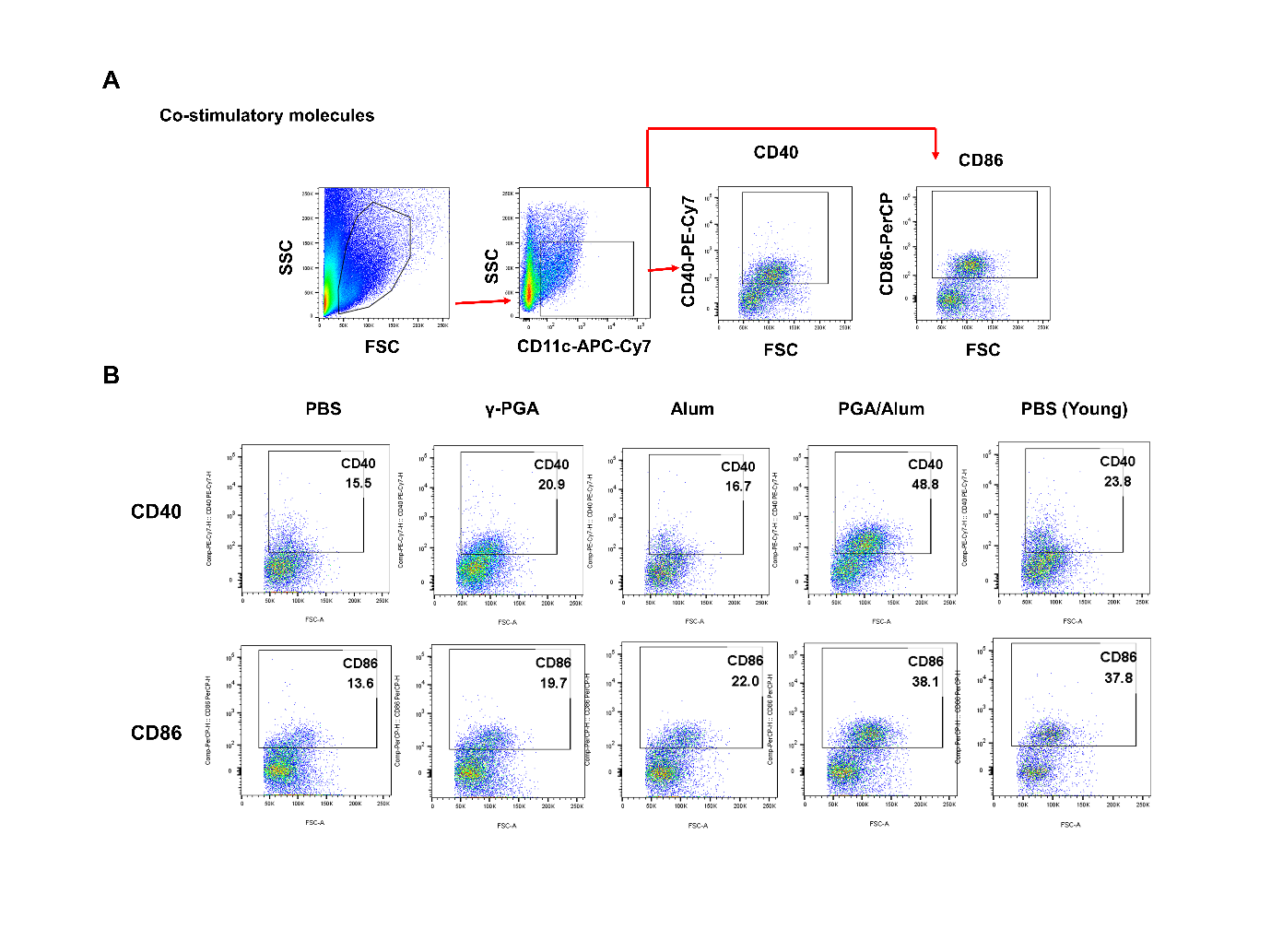


**Supplementary Fig. 4** Flow cytometry strategy for analyzing expression of co-stimulatory molecules on DCs. Gating strategy of CD40 and CD80 on CD11c^+^ DCs and representative dot plots in each group. Numbers in representative dot plots indicate the percentage of positive cells.


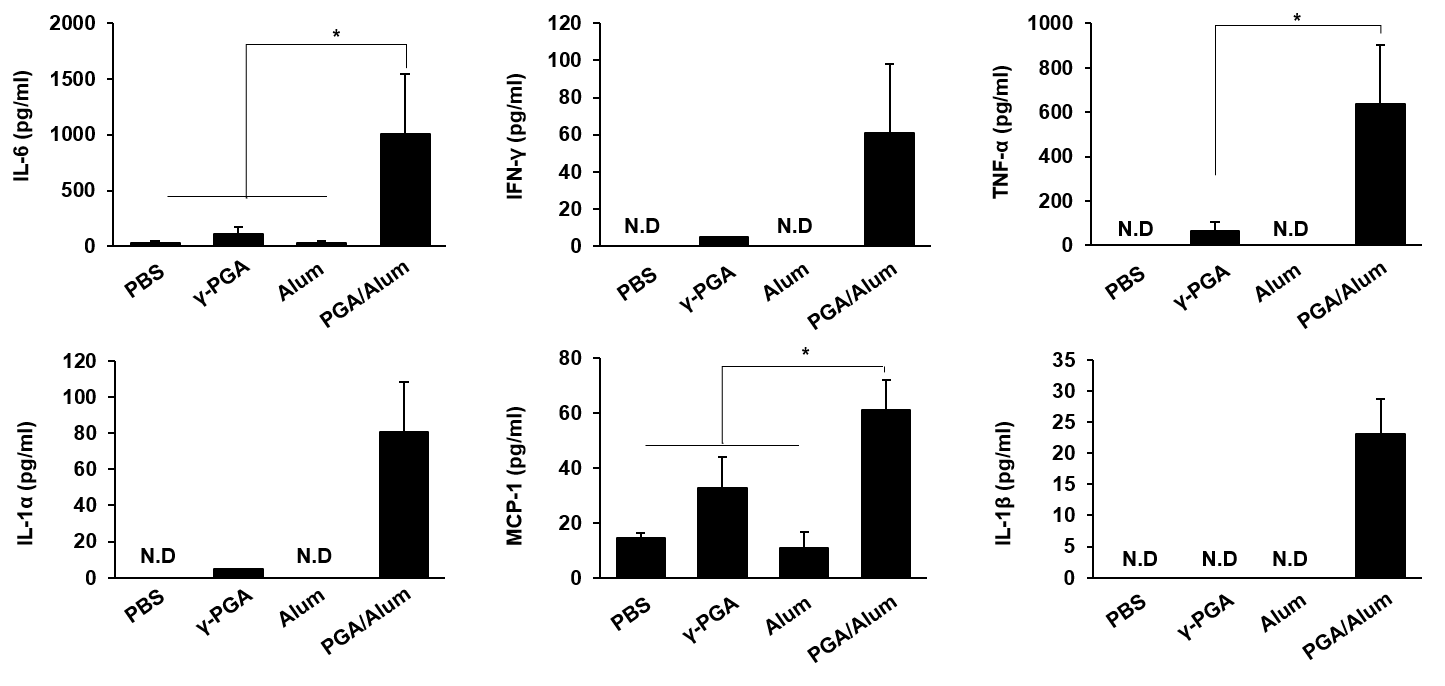


**Supplementary Fig. 5** PGA/Alum robustly induces the production of inflammatory cytokines in DCs from the aged mice. Splenic DCs were isolated from aged mice and stimulated with 100 μg/mL of γ-PGA, 100 μg/mL of alum, or 200 μg/mL of PGA/Alum for 30 h. Levels of the cytokines were determined in the culture supernatants using a Legendplex immunoassay kit. Statistical significance was analyzed by one-way ANOVA/Bonferroni; **P* < 0.05. N.D, not-detected.


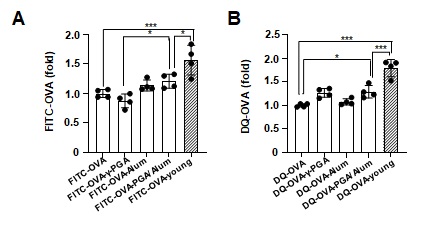


**Supplementary Fig. 6** PGA/Alum increases antigen uptake and processing on DCs from the aged mice. CD11c^+^ DCs were purified from splenocytes of young and aged mice (n = 4 per group) and then incubated with FITC-OVA (**A**) or DQ-OVA (**B**) alone or combined with 100 μg/mL of γ-PGA, 100 μg/mL of alum, or 200 μg/mL of PGA/Alum for 6 or 18 h, respectively. Percentages of the FITC-OVA^+^ DCs and DQ-OVA^+^ DCs were analyzed via flow cytometry and the fold-change values were calculated in comparison with FITC-OVA or DQ-OVA alone-treated cells of the aged mice. Statistical significance was analyzed by one-way ANOVA/Bonferroni; **P* < 0.05, ***P* < 0.01, and *** *P* <0.001.


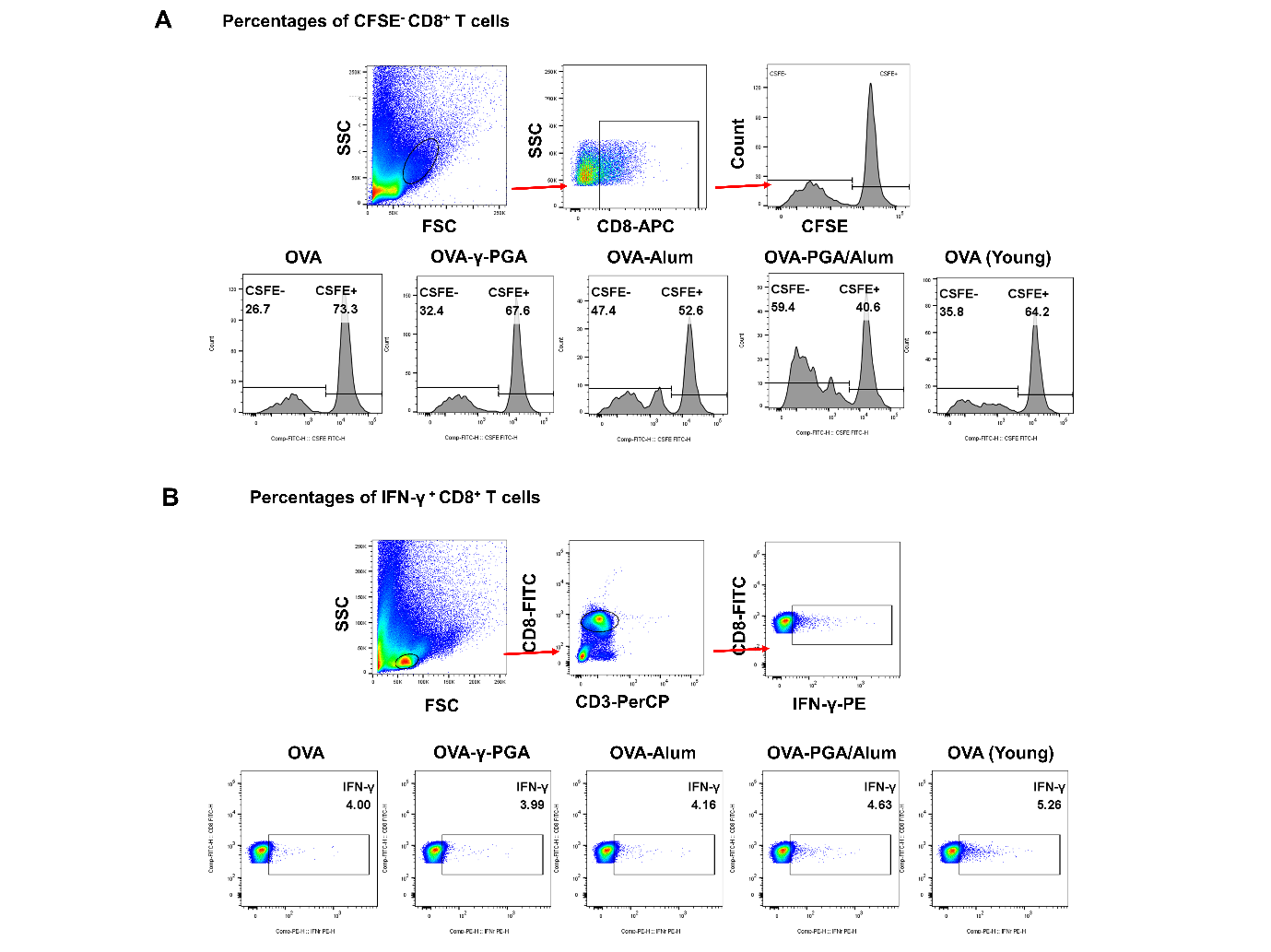


**Supplementary Fig. 7** Flow cytometry strategies for analyzing CFSE^-^ CD8^+^ T cells and IFN-γ^+^ CD8^+^ T cells in splenocytes. (**A**) Gating strategy of CFSE-labeled CD8^+^ T cells and representative histograms in each group. Numbers above the bracketed lines in representative histograms indicate the percentage of CFSE^-^ (proliferated) and CFSE^+^ cells. (**B**) Gating strategy of IFN-γ^+^CD8^+^CD3^+^ T cells and representative dot plots in each group. Numbers in representative dot plots indicate the percentage of positive cells.


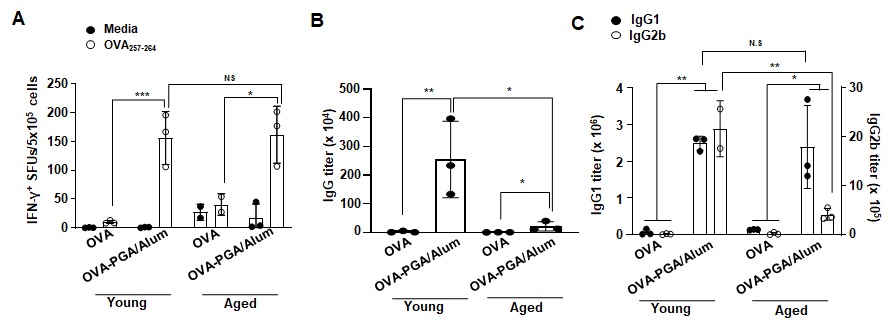


**Supplementary Fig. 8** PGA/Alum enhances antigen-specific cellular immune response of the aged mice as much as the young mice rather than humoral immune response. Young and aged mice (n = 3 per group) were i.m. immunized with OVA alone or mixed with PGA/Alum on days 0, 14, and 21. Seven days after last immunization, the splenocytes and sera were obtained. (**A**) The splenocytes were stimulated with 1 μg/mL of OVA_257-264_ peptide for 60 h, and IFN- γ^+^ SFUs were detected via ELISPOT assays. (**B** and **C**) ELISA was performed to determine antibody titers of OVA-specific IgG (B), IgG1, and IgG2b (C). Statistical significance was analyzed by one-way ANOVA/Bonferroni; **P*<0.05, ***P*<0.01, and ****P*<0.001. N.S, not significant.

**
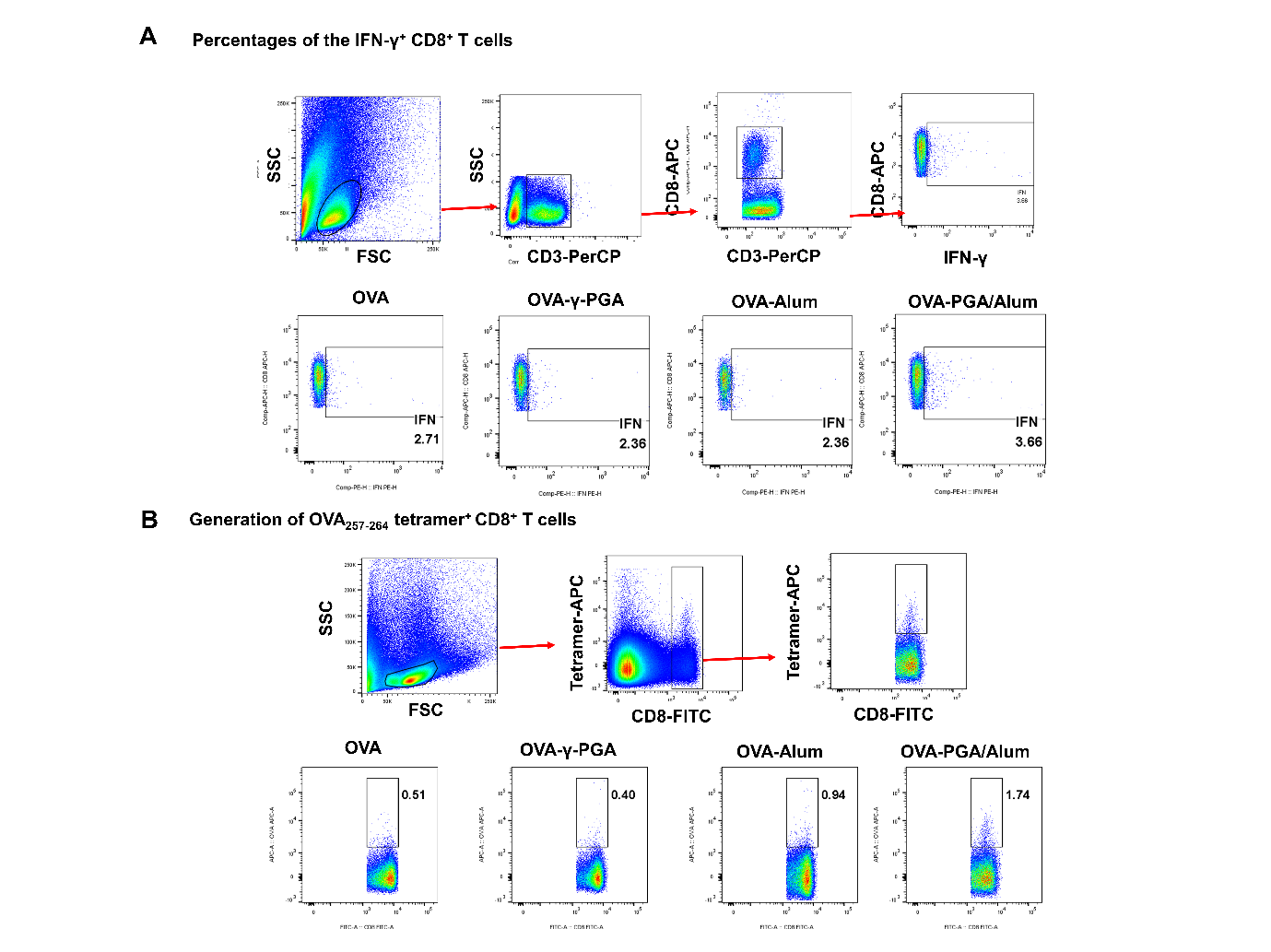
**

**Supplementary Fig. 9** Flow cytometry strategies for analyzing IFN-γ^+^ CD8^+^ T cells and OVA_257-624_ tetramer^+^ CD8^+^ T cells in splenocytes. (**A**) Gating strategy of IFN-γ^+^CD8^+^CD3^+^ T cells and representative dot plots in each group. Numbers in representative dot plots indicate the percentage of positive cells. (**B**) Gating strategy of OVA_257-624_ tetramer^+^ CD8^+^ T cells and representative dot plots in each group. Numbers in representative dot plots indicate the percentage of OVA_257-624_ tetramer^+^ and OVA_257-624_ tetramer^-^ cells.

**
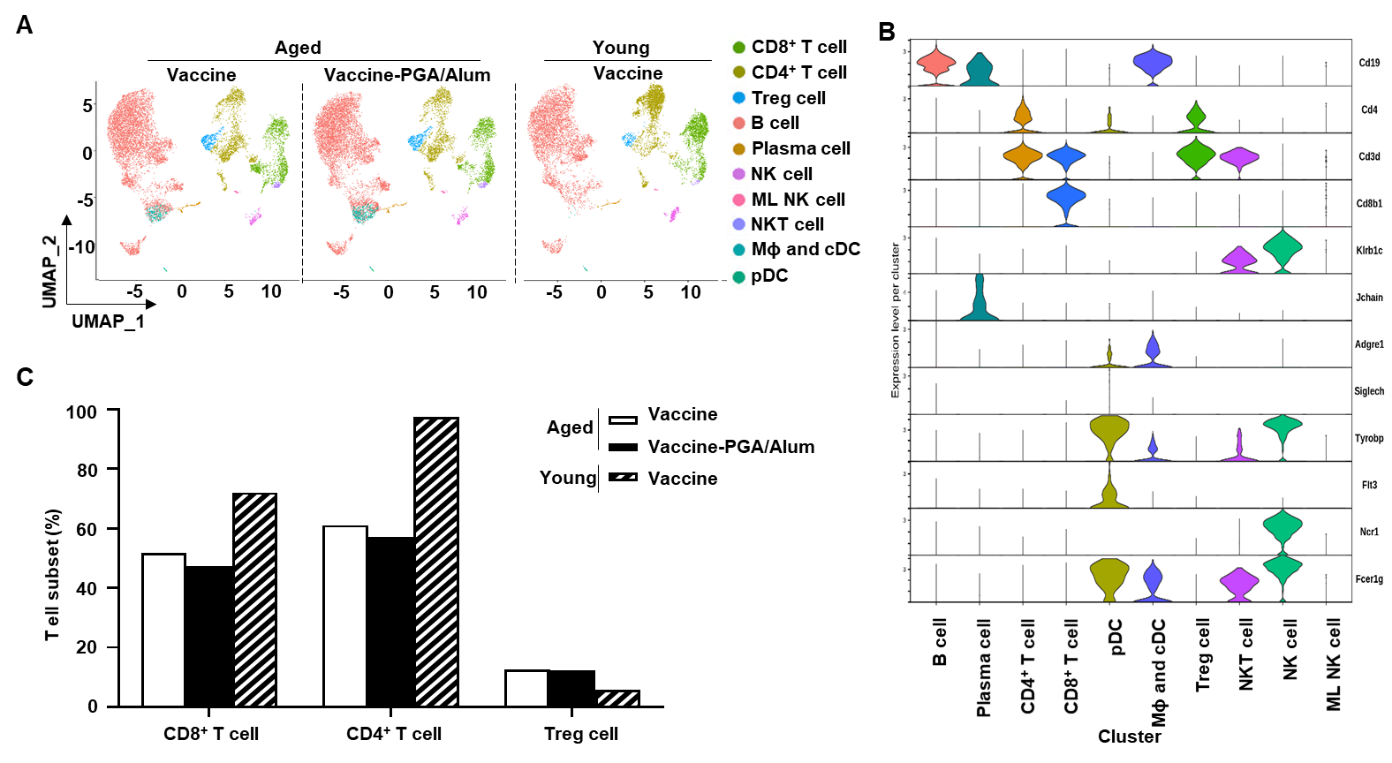
**

**Supplementary Fig. 10** Immune cell landscape of the aged mice immunized with vaccine alone or combined with PGA/Alum. (**A**) UMAP dimensionality reduction embedding CD45^+^ cells isolated from the splenocytes from aged (pool of n = 3, 18-month-old) mice immunized with vaccine (10,042 cells) or vaccine-PGA/Alum (11,212 cells) and vaccine-immunized young (pool of n = 3, 6-week-old, 10,042 cells). (**B**) Violin plots showing scaled expression levels of marker genes per cluster that are used to select clusters for the downstream analysis. (**C**) The proportions of T cell subsets in the annotated clusters. NK, natural killer. ML NK, memory-like NK. NKT, natural killer T. Mɸ, macrophage. cDC, conventional dendritic cell. pDC, plasmacytoid dendritic cell.**
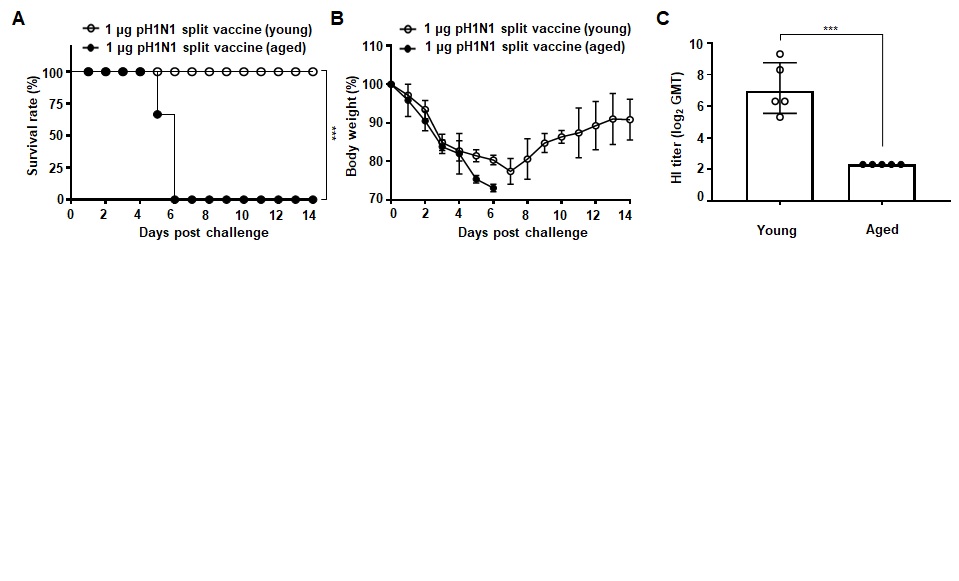
**

**Supplementary Fig. 11** None of the aged mice are protected against the pH1N1 virus infection despite immunization of the pH1N1 split vaccine. Young (6-week-old) and aged (18-month-old) mice were i.m. administered the pH1N1 split vaccine antigen on days 0 and 14. (**A, B**) Two weeks after the final administration, the vaccinated mice were i.n. challenged with 50 LD_50_ pH1N1 virus. Survival rates (**A**) and body weight changes (**B**) were monitored for up to 14 days post-challenge. Each data point represents an average percentage. Statistical significance was analyzed by log-rank test (A); ****P*<0.001.


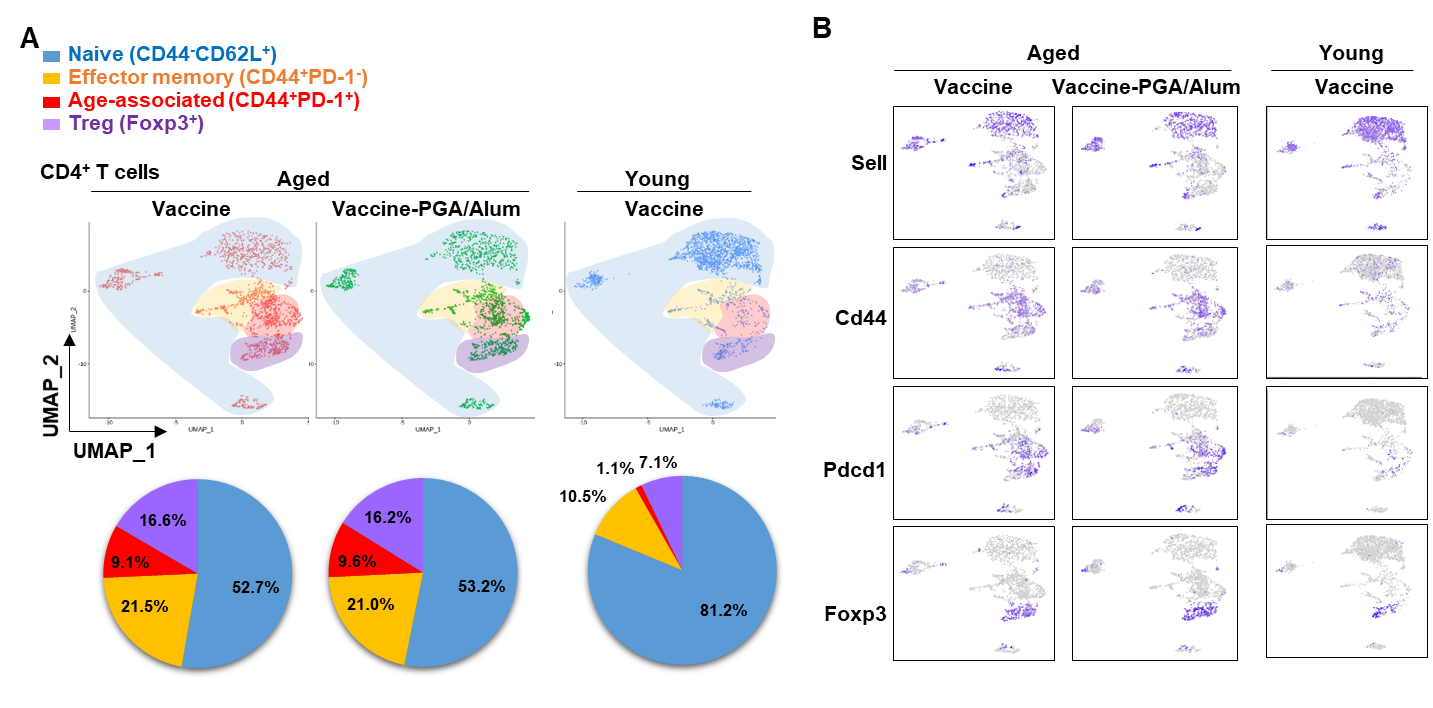


**Supplementary Fig. 12** Effects of PGA/Alum on CD4+ T cell populations in the influenza vaccine-immunized aged mice. (**A**) UMAP dimensionality reduction embedding CD4^+^ T cells within CD45^+^ cells isolated from the splenocytes from the aged (pool of n = 3, 18-month-old) mice immunized with vaccine (1,878 cells) or vaccine-PGA/Alum (1,836 cells), and vaccine-immunized young (pool of n = 3, 6-week-old, 1,793 cells) and differential representation of CD4^+^ T cell subset. (**B**) Scaled gene expression of CD4^+^ T cells.


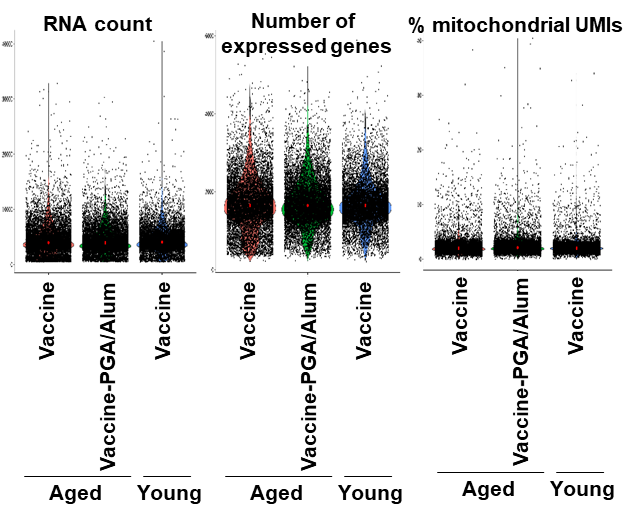


**Supplementary Fig. 13** Quality control for analyzing scRNA-seq and immune profiles. RNA count, number of expressed genes, and percentage of mitochondrial UMI per scRNA-seq sample.
